# Effects of Minocycline on Urine Albumin, Interleukin-6, and Osteoprotegerin in Patients with Diabetic Nephropathy: A Randomized Controlled Pilot Trial

**DOI:** 10.1371/journal.pone.0152357

**Published:** 2016-03-28

**Authors:** Anuja P. Shah, Jenny I. Shen, Ying Wang, Lili Tong, Youngju Pak, Ali Andalibi, Janine A. LaPage, Sharon G. Adler

**Affiliations:** Los Angeles Biomedical Research Institute at Harbor-UCLA Medical Center, 1124 West Carson Street, Torrance, CA, 90509, United States of America; Kaohsiung Medical University HospitalKaohsiung Medical University HospitalKaohsiung Medical University Hospital, TAIWAN

## Abstract

**Background:**

We tested minocycline as an anti-proteinuric adjunct to renin-angiotensin-aldosterone system inhibitors (RAASi) in diabetic nephropathy (DN) and measured urinary biomarkers to evaluate minocycline’s biological effects.

**Methods:**

**Design**: Prospective, single center, randomized, placebo-controlled, intention-to-treat pilot trial. **Inclusion**. Type 2 diabetes/DN; Baseline creatinine clearance > 30 mL/min; proteinuria ≥ 1.0 g/day; Age ≥30 years; BP <150/95 mm Hg; intolerant of/at maximum RAASi dose. **Protocol**. 3-wk screening; Baseline randomization; Urine and blood measures at months 1, 2, 4, and Month 6 study completion. Urine interleukin-6 (IL-6) and osteoprotegerin were measured in a subset. **Primary outcome**. Natural log of urine protein/creatinine (ln U P:Cr) ratio at Month 6 vs Baseline.

**Results:**

30 patients completed the study. The 15% decline in U P: Cr in minocycline patients (6 month P:Cr ÷ Baseline P:Cr, 0.85 vs. 0.92) was not significant (p = 0.27). Creatinine clearance did not differ in the 2 groups. Urine IL-6:Cr (p = 0.03) and osteoprotegerin/Cr (p = 0.046) decrements were significant. Minocycline modified the relationship between urine IL-6 and proteinuria, suggesting a protective biological effect.

**Conclusions:**

Although the decline in U P:Cr in minocycline patients was not statistically significant, the significant differences in urine IL-6 and osteoprotegerin suggest that minocycline may confer cytoprotection in patients with DN, providing a rationale for further study.

**Trial Registration:**

Clinicaltrials.gov NCT01779089

## Introduction

Diabetic nephropathy (DN) remains a major source of mortality and morbidity in patients with Type 2 diabetes (DM). Although incident end stage renal disease (ESRD) from DN is declining, prevalence continues to rise (www.usrds.org). While angiotensin converting enzyme inhibitors (ACEi) and angiotensin receptor blockers (ARBs) slow progression to ESRD, they do not regress or arrest progression to ESRD. Combining drugs that inhibit multiple targets in the renin-angiotensin-aldosterone system induce unacceptably high rates of acute kidney injury and hyperkalemia [1–3]. Recent studies testing drugs with treatment targets outside of the renin-angiotensin-aldosterone system, including bardoxolone, ruboxistaurin, pimagedine, monoclonal anti-connective tissue growth factor antibody, and sulodexide either failed to demonstrate benefit, were unsafe, or were abandoned for economic reasons[4–8]. A safe and inexpensive medication whose therapeutic target lies outside of the renin-angiotensin-aldosterone pathway would be useful as adjunctive therapy for DN.

Minocycline is a semi-synthetic tetracycline whose broad cytoprotective properties have lately become apparent[9]. Minocycline attenuated diabetic nephropathy and diabetic retinopathy in animal models[10, 11]. In two small clinical trials, tetracycline reduced proteinuria at least transiently in patients with DN [12, 13]. This pilot study was undertaken to assess the safety and efficacy of minocycline as an anti-proteinuric agent in patients with type 2 DM with proteinuria and eGFR ≥ 30 ml/min/1.73 m^2^ who were either unable to tolerate renin-angiotensin-aldosterone system inhibitors (RAASi) or who were already taking an ACEi or ARB at a maximally tolerated dose. This study also sought biological evidence of minocycline activity by comparing the change in urine biomarkers interleukin-6 (IL-6) and osteoprotegerin in the 6-month treatment period across the randomized comparison.

## Materials and Methods

### Design

This proof of concept pilot study employed a prospective, single center, randomized, placebo-controlled design. Patients were randomized in two strata based on the mean 24 hour urine protein excretion at baseline, as follows: (a) < 3 g/24h and (b) ≥ 3 g/24h. The study consisted of a 3-week screening period; a baseline visit (Day 0) during which the patients were randomly assigned to the active drug (minocycline 100 mg by mouth twice a day) or to matching placebo; a 24-week treatment period; a study completion visit at week 24; and a 2-month post-completion washout period with repeat urine and blood measures.

**Inclusion criteria** were clinical diagnosis of Type 2 DM and DN as described in the Family Investigation of Nephropathy and Diabetes Protocol[14]; baseline creatinine clearance ≥ 30 mL/min/1.73 m^2^ (at first screening visit); proteinuria ≥ 1.0 g/day (at first screening visit); age ≥30 years; blood pressure at baseline <150/95 mm Hg (measured sitting after 10 min rest at first screening visit); and adequate hepatic function defined as total bilirubin < 1.5 x the upper limit of the normal range (ULN), AST (SGOT) and ALT (SGPT) < 2.5 x ULN. Patients taking ACEi, ARBs, aliskerin, spironolactone and/or diltiazem could be entered, but dosing was not to change during the period of study or within 1 month prior to the first of the baseline proteinuria measurements. There was an effort to selectively recruit patients who were intolerant of ACEi or ARBs at any dose due to hyperkalemia, as well as those who had reached a maximum dose defined either by the development of hyperkalemia or by the manufacturers’ suggested dose.

**Exclusion criteria** were non-steroidal anti-inflammatory drug (NSAID, including COX-2 inhibitors) use > 3 pills/week habitually; diagnosis of neurodegenerative diseases (Parkinson's disease, Huntington’s disease, multiple sclerosis, Alzheimer's disease, etc.); any unstable medical illness (unstable angina, advanced cancer, etc.) over the last 30 days; history of liver disease; history of hematologic disease (screening white blood cell count less than 3,800/mm^3^); history of systemic vasculitis or systemic lupus erythematosus; treatment with procainamide or hydralazine; history of vestibular disease (excluding benign position vertigo); pregnancy or lactation; allergy to tetracycline antibiotics; use of minocycline within 30 days of baseline visit; use of anti-epileptic medications other than gabapentin; use of lithium, digoxin, warfarin, other anticoagulants, and theophylline; limited mental capacity rendering the subject unable to provide written informed consent or comply with evaluation procedures; history of recent alcohol or drug abuse or noncompliance with treatment or other experimental protocols; and/or use of any investigational drug within 30 days prior to the baseline visit. Women with the potential to become pregnant who were not willing to practice double-barrier birth control were also excluded.

A sample size of 52 patients per treatment group was estimated sufficient to detect a 35% decrease in 24 hour urine protein in the minocycline group compared to the placebo group, assuming that the data is analyzed on a log-scale [ln(6MonthP:Cr)—ln(baseline P:Cr) in placebo vs. minocycline group], with 90% power, using a one-sided independent t-test at a 0.025 alpha level and an intra subject coefficient of variation of 50%. Patients were recruited from July 1, 2009 to June 30, 2012 and the date of the last subject visit was April 2, 2013.

### Randomization and medication administration

Minocycline and placebo were prepared by a compounding pharmacy (Rite Price Pharmacy, Inc., Whittier, CA, 90603), which provided each in an identical red capsule. Medications were stored in the research pharmacy at the Los Angeles Biomedical Research Institute (LA, CA). The research pharmacist maintained the study medications and dispensed them according to randomization tables created by the PROC PLAN randomization module in SAS. Patients were randomized in two strata based on the baseline 24 hour urine protein excretion (< 3 g/24h and ≥ 3 g/24h). Study personnel and patients were blinded to the study medication assignment until the close of data cleaning.

As originally planned, the minocycline dose was 100 mg orally twice daily. Recommendations for adjusting minocycline dose for renal function range from no change to dose reduction by an undefined amount. The first 3 subjects complained of dizziness and nausea. In response, the blind was maintained, and with IRB approval, the dosing protocol was modified. The dose was lowered to 100 mg daily in these three patients, and this dose was tolerated. For all subsequent patients, dosing began at 100 mg daily in month 1; the dose was increased as tolerated. Adherence to the study prescription was assessed by pill counting at every visit. Adherence was reported as the % of prescribed tablets taken by the subject over the course of the 6-months, taking into account the dose reductions necessitated by reported symptoms. Adherence was not defined in a binary fashion.

### Laboratory measurements

Unless stated otherwise, all laboratory measurements were made in the CLIA-approved laboratory of the Harbor-UCLA Medical Center. Measurements for electrolytes, blood urea nitrogen, serum creatinine, and liver function tests were performed by an automated analyzer (Beckman Coulter UniCelDxC800 Synchron Clinical System). Anti-nuclear antibody (ANA, Zeus Scientific Helmed Processor) and anti-neutrophil cytoplasmic antibody (ANCA, Quest Diagnostics, West Hills, CA) tests were performed as part of the safety analyses. Urine protein and creatinine were measured using a Beckman Coulter UniCelDxC800 Synchron Clinical System. Urine for interleukin-6 (IL-6) and osteoprotegerin were collected at baseline and month 6, measured by electrochemiluminescence at Meso Scale Discovery (Rockville, MD), expressed as a ratio with urine creatinine, and the results of the Baseline and end-of-treatment (Month 6) values compared.

### Safety and tolerability assessments

Subjects were questioned at each clinic visit regarding new symptoms and/or health changes since the prior visit, and all responses were collected, tabulated, and reported as adverse events to the sponsor and to our local IRB using Common Terminology Criteria for Adverse Events v3.0 (CTCAE). Subjects were instructed to notify the center if hospitalized, and these were reported as serious adverse events to the sponsor and to the local IRB. Safety assessments additionally consisted of documenting concomitant medications and/or therapies; monitoring lab parameters including CBC, blood chemistries (including renal function, liver function, electrolytes, serum albumin), serum β-HCG as appropriate, vital signs including blood pressure and weight, and assessments for edema. ANA and ANCA titers were measured at baseline, and at 2 and 6 months to ascertain for subclinical systemic lupus erythematosus or vasculitis. Each is a rare complication of minocycline therapy [15, 16]. Criteria for the evaluation of safety included a comparison of pre-treatment to during-treatment laboratory tests; assessment of the occurrence, severity, and duration of all adverse events and serious adverse events, with the investigator’s assessment of causality; and a comparison of pre- and post-treatment physical examinations.

### Statistical analysis

We described baseline characteristics of the cohort using means and standard deviations for normally distributed continuous data, medians and 25th and 75th percentile values for non-normally distributed data, and counts and proportions for categorical data. Differences between the placebo and minocycline groups were compared using the Student’s t test, Wilcoxon rank-sum test, or χ^2^ test, as appropriate.

The pre-defined primary outcome was change from baseline to month 6 in the 24 hour urine protein/creatinine (average of two collections per time point), analyzed on a log-scale [ln(6MonthP:Cr)-ln(baseline P:Cr)]. One-tailed two-sample t-test was used to compare the outcome between the placebo and minocycline groups as it was normally distributed (p-value for the Shapiro-Wilk test was 0.31). We also analyzed the primary outcome on the log-scale using a linear model with treatment as factor and the Baseline 24 hour urine protein as a covariate to ensure that differences in baseline proteinuria did not substantially alter the results. The ratio of the change in P:Cr from Baseline to Month 6 between the minocycline and placebo groups and the corresponding two-sided 95% confidence interval were transformed back to the original scale.

Secondary outcomes included changes in urine P:Cr in the daytime, nighttime, and a random spot collection, and urine albumin: creatinine ratio in the daytime, overnight, complete, and a random spot collection. We also analyzed change from baseline to month 6 in the creatinine clearance (CrCl), analyzed on a log-scale [ln(6Month CrCl)-ln(baseline CrCl)]. We further compared the percent change in urine IL6/creatinine and urine osteoprotegerin/creatinine from Baseline to Month 6. Two-tailed two-sample t-tests were used to compare the outcomes between the placebo and minocycline groups for all of the secondary outcomes except for the percent change in urine osteoprotegerin, for which we used the Wilcoxon rank-sum test since the osteoprotegerin data were non-normally distributed.

We also used linear regression to assess for correlation between the primary outcome and the percent change in biomarkers. To look for potential changes in the effect of minocycline on proteinuria over time, we used a random intercept mixed effects model for repeated measures with unstructured covariate matrix and baseline 24 hour urine protein as a covariate. We similarly looked for a potential change in the decline of creatinine clearance over time between the minocycline and placebo groups using a random intercept mixed effect model with unstructured covariate matrix and baseline creatinine clearance as a covariate.

P-value<0.025 was considered statistically significant for the primary outcome. For all other outcomes we considered p-values <0.05 to be significant. We did not adjust for multiple comparisons as this is a pilot study and we consider these analyses to be exploratory. All analyses were performed using SAS Enterprise Guide 4.3 (SAS Institute Inc., Cary, NC, USA).

The study was approved by and conducted under the oversight of the Institutional Review Board of the Los Angeles Biomedical Research Institute Compliance Office and the UCLA Clinical Science and Translational Institute on February 10, 2009, and is listed with ClinicalTrials.gov (NCT01779089). The study was registered after enrollment had started because there was a miscommunication; we believed our institution had registered the study, and when we found out they had not, the investigators registered it themselves. Written consent was obtained from all participants. The authors confirm that all ongoing and related trials for this drug are registered.

## Results

### Study population baseline characteristics

**Fig 1:**
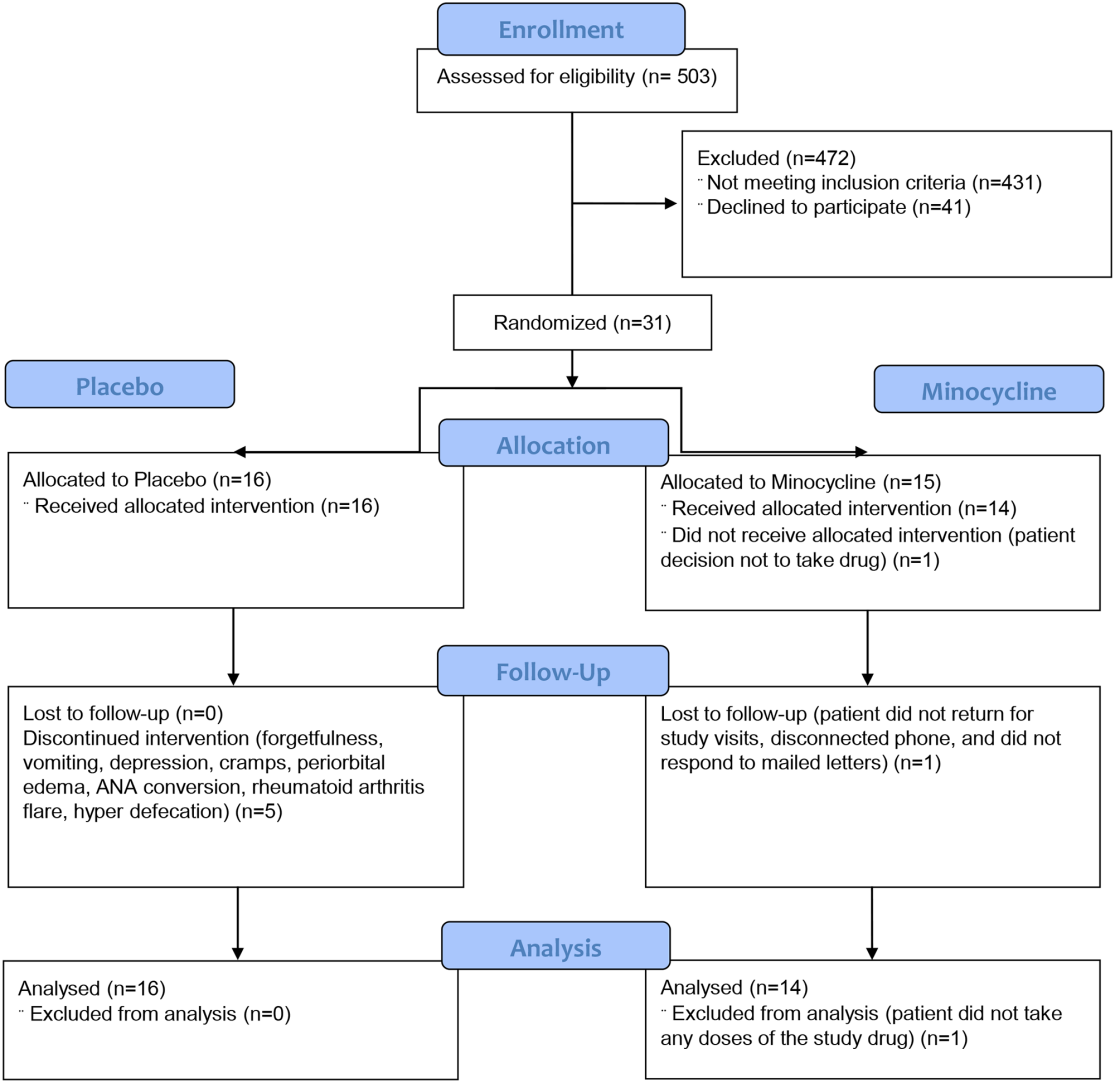
Enrollment of subjects.

Patients were recruited from July 1, 2009 to June 30, 2012. Of the 54 subjects who had a screening visit, 31 were randomized (Fig 1). The reasons for failure after screening were: Creatinine clearance too low (n = 8); compliance issues during screening (n = 8); abnormal liver function tests (n = 1); ANA positive (n = 3); claudication (n = 1); and blood pressure out-of-control (n = 1). Recruitment was suspended at the end of grant funding before full recruitment was accomplished. Per protocol, a disproportionate number of subjects who were entered were not taking any RAASi: 17 due to hyperkalemia, 1 due to angioedema, and 1 due to combined hyperkalemia, hypotension, and acute kidney injury. One subject signed the consent form but withdrew immediately before taking any study medication; this subject was omitted from the analysis. Of the remaining 30 subjects, the clinical characteristics at baseline were evenly distributed by age, gender, ethnicity, diabetes duration, baseline systolic and diastolic blood pressures, proteinuria, and creatinine clearance and not significantly different with respect to statin and RAASi use. HbA1c in the minocycline group was higher than in the placebo group (8.4% (7.5–8.9%) vs 7.3% (6.5–8.0%), p = 0.05) (Table 1). There were no significant clinical differences between the two arms at the end of the trial, either (Table 2). In the minocycline-assigned group, 12 of 14 subjects tolerated the full twice-daily dose; 2 required a dose reduction. In the placebo-assigned group, 13 of the 15 subjects tolerated the twice-daily dose; 2 required a dose reduction. There was no difference in adherence in the two arms as measured by pill counting. The trial was ended after 6 months, as pre-specified in the protocol. The date of the last subject visit was April 2, 2013.

**Table 1:** Baseline characteristics of the randomized groups.

Variable	Minocycline (n = 14)	Placebo (n = 16)
Age	51 (41–62)	52 (30–68)
Sex (% male)	79%	69%
Race		
Black	1	1
Hispanic	10	14
Asian/South Pacific	3	1
Diabetes duration (years)	14±7	13±6
Baseline HgbA1c, %	8.5±0.6	7.7±0.4
Baseline mean arterial pressure (mm Hg)	97±2	94±2
% on RAASi*	29%	44%
% on Vitamin D3 analog	0%	0%
% on Statin	86%	56%
Baseline Urine protein/creatinine (mg/g)		
24 hour	2.67±0.21	4.61±0.91
daytime	2.79±0.23	4.6±1.01
nighttime	2.48±0.22	4.17±0.8
Baseline Urine microalbumin/creatinine (MACR) (mg/g)		
24 hour	1969±182	2891±586
daytime	2086±204	3215±707
nighttime	1788±171	2847±532
Baseline creatinine clearance (ml/min/1.73 m^2^)	70±10	53±6

*RAASi = renin-angiotensin-aldosterone system inhibitors

**Table 2:** Subject characteristics at 6 months.

Variable	Minocycline (n = 14)	Placebo (n = 16)
6 month HbA1c, %	8.5±1.7	7.5±1.2
6 month mean arterial pressure, mm Hg	96 ±12	99±14
% on RAASi*	29%	44%
% on Vitamin D3 analog	14%	25%
% on Statin	43%	50%
6 month Urine Protein/Creatinine (mg/g)		
24 hour	2.46±1.47	4.25±4.35
Daytime	2.49±-1.6	4.50±4.58
Nighttime	2.42±1.4	4.12±4.2
6 month Urine microalbumin/creatinine (MACR) (mg/g)		
24 hour	2061±1205	2809±2677
Daytime	2601±2808	2976±2842
nighttime	1766± 1111	2693±2597
6 month creatinine clearnace (ml/min/1.73 m^2^)	63± 34	46 ±29
6 month Pill Count (adherence)	81 + 19%	76 + 20%

*RAASi = renin-angiotensin-aldosterone system inhibitors

### Primary outcome

#### Change in urine protein to creatinine

**Fig 2:**
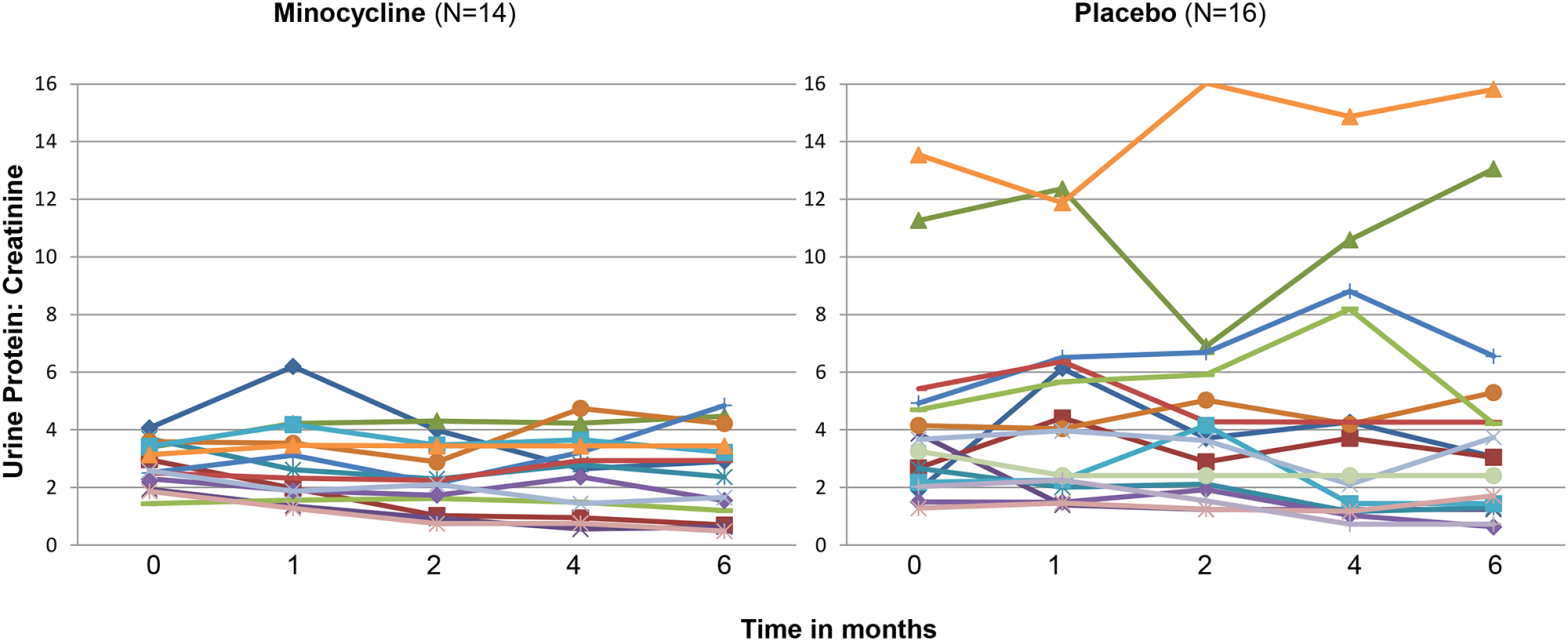
Spaghetti plots of the urine protein: creatinine measurements of each patient in the study over the course of the trial.

**Fig 3:**
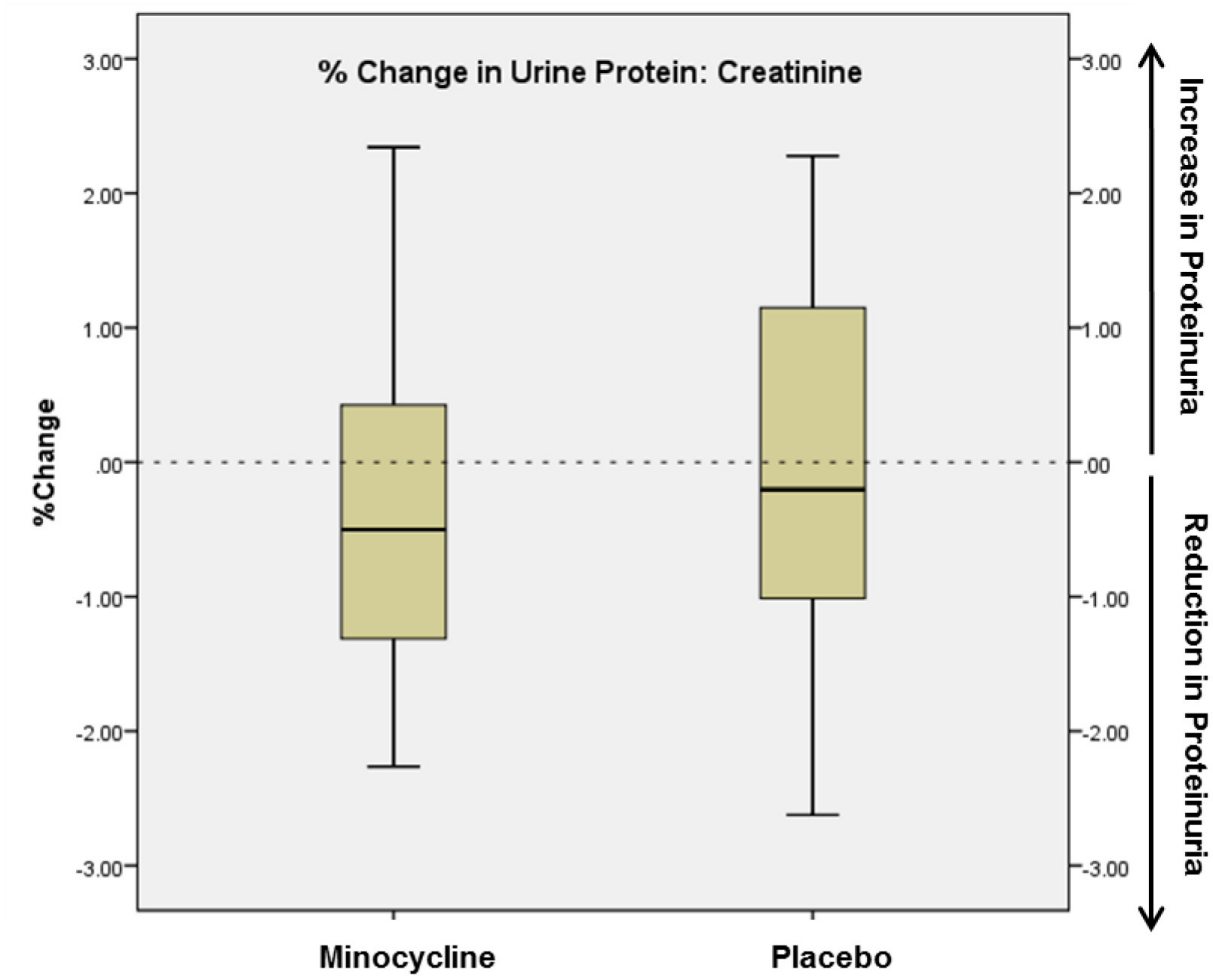
Percent change in 24-hour urine protein/creatinine measurements from baseline to 6 months in placebo and minocycline groups. The edges of the boxes represent the 25% and 75% percentiles, the middle lines represents the median, and the whiskers extend to the minimum and maximum values. The primary outcome, ln (6Month P:Cr/baseline P:Cr), was not significantly different between the groups (p = 0.27).

Proteinuria and albuminuria declined in both the minocycline and the placebo groups (Fig 2). The ratio of the change in P:Cr from Baseline to Month 6 between the minocycline and placebo groups was 0.87 (95% CI 0.53–1.43). Although the decline in proteinuria was numerically more in the minocycline than the placebo group (mean percent change -15% vs. -8%), the difference was not statistically significant (Fig 3).

### Secondary assessments

#### Change in proteinuria

From the mixed effects model, the minocycline group had less estimated proteinuria than the placebo group at every follow-up study time point (1, 2, 4, and 6 months), even after adjustment for baseline proteinuria; however, these differences were not statistically significant (Table 3). Of the 16 patients randomized to placebo, 50% (n = 8) had a decrement in proteinuria during the course of the study. Of the 14 patients randomized to minocycline, 64% (n = 9) had a decrement in proteinuria (NS). Change in proteinuria did not correlate with baseline proteinuria, creatinine clearance, or eGFR. In subjects in the minocycline group in whom a decrement in proteinuria occurred, the decrease was first evident between months 2 and 4 of treatment. In the majority, the slope of the decline in proteinuria had not yet plateaued by the Month 6 end-of-study visit (data not shown), suggesting, but not proving, that additional exposure to medication may have lowered the proteinuria more.

**Table 3:** Ratio of proteinuria in minocycline vs. placebo group, adjusted for baseline proteinuria.*

Time point	Estimated urine P:Cr in the minocycline group / Estimated urine P: Cr in the placebo group	P-value
1 month	0.90	0.58
2 months	0.79	0.21
4 months	0.94	0.73
6 months	0.90	0.56

*Estimated from a random intercept mixed-effects model for repeated measures.

There was no statistically significant difference in proteinuria or albuminuria between the two groups in the daytime, nighttime, or random spot collections. We also did not find any difference in proteinuria or albuminuria from baseline to washout, which was two months post-discontinuation of the drug, or from 6 months to washout.

#### Urine IL-6/creatinine and urine osteoprotegerin/creatinine levels

**Fig 4:**
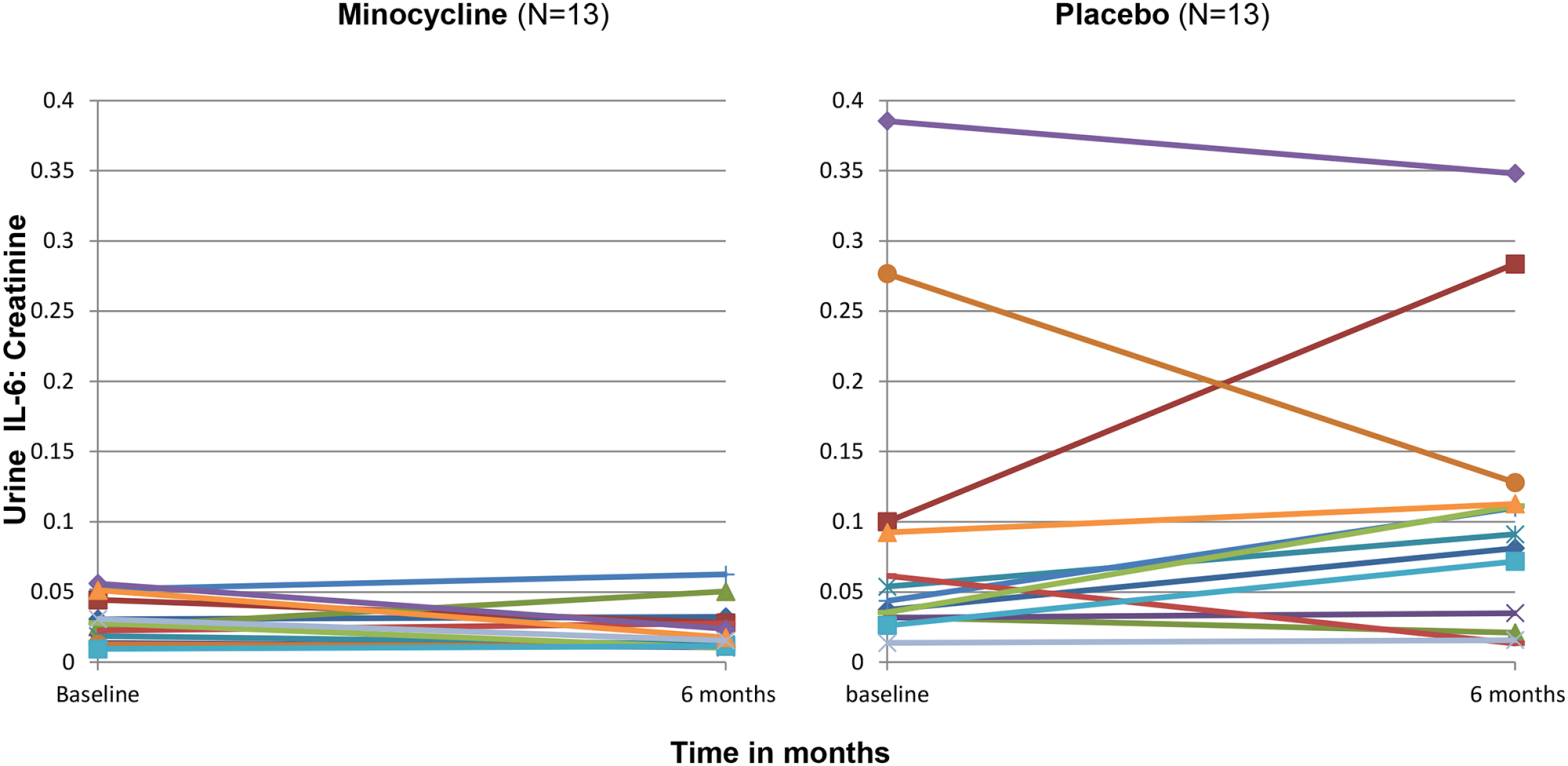
Spaghetti plots of the urine IL6: creatinine measurements of each patient in the study from baseline to 6 months (end of trial). Baseline urine values were not available for 3 subjects.

**Fig 5:**
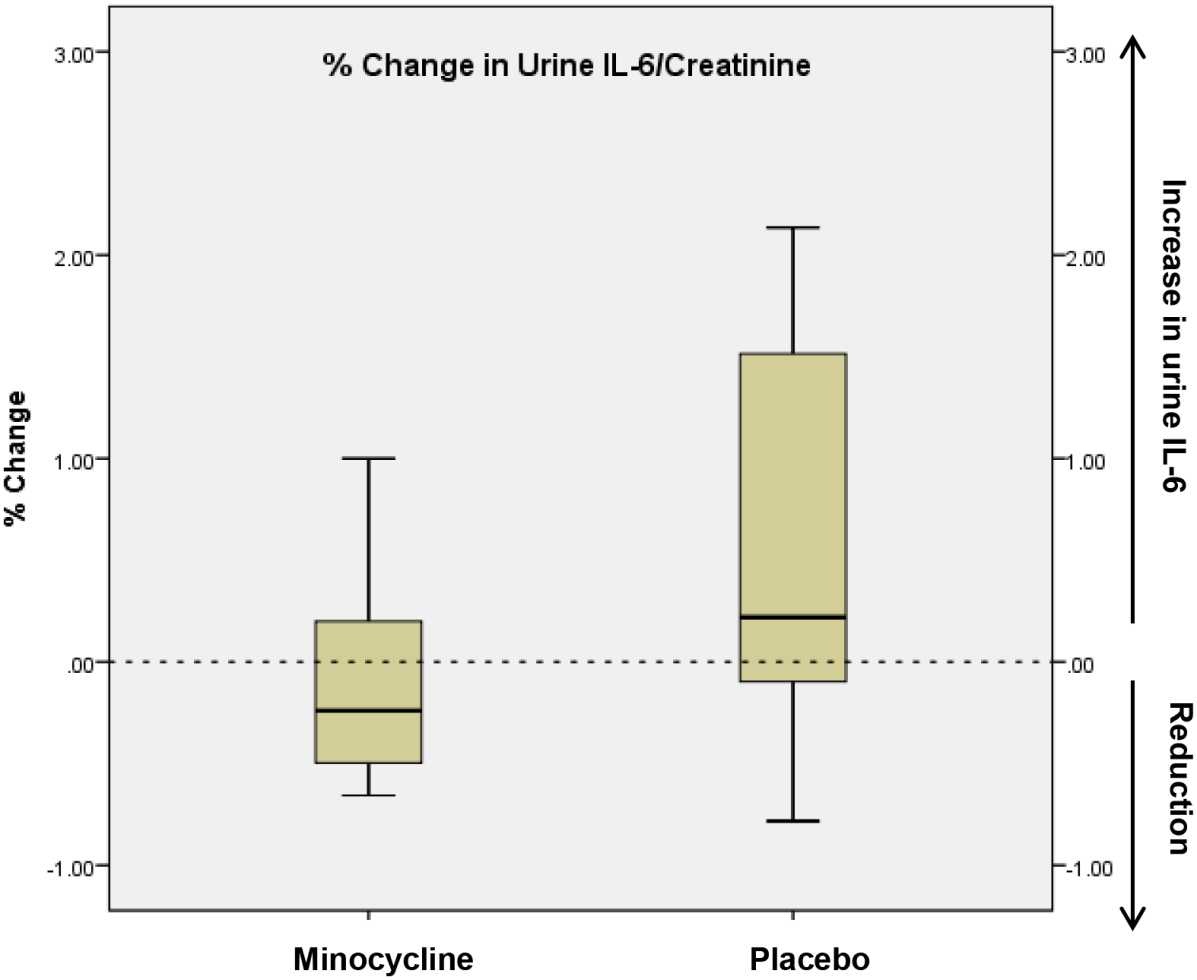
% change in 24-hour urine Il-6: creatinine measurements from baseline to 6 months in placebo and minocycline groups. The edges of the boxes represent the 25% and 75% percentiles, the middle lines represents the median, and the whiskers extend to the minimum and maximum values. The %change in urine IL-6: creatinine was significantly lower in the minocycline group vs. the placebo group (mean % change -0.13 vs. 0.60, P = 0.03).

**Fig 6:**
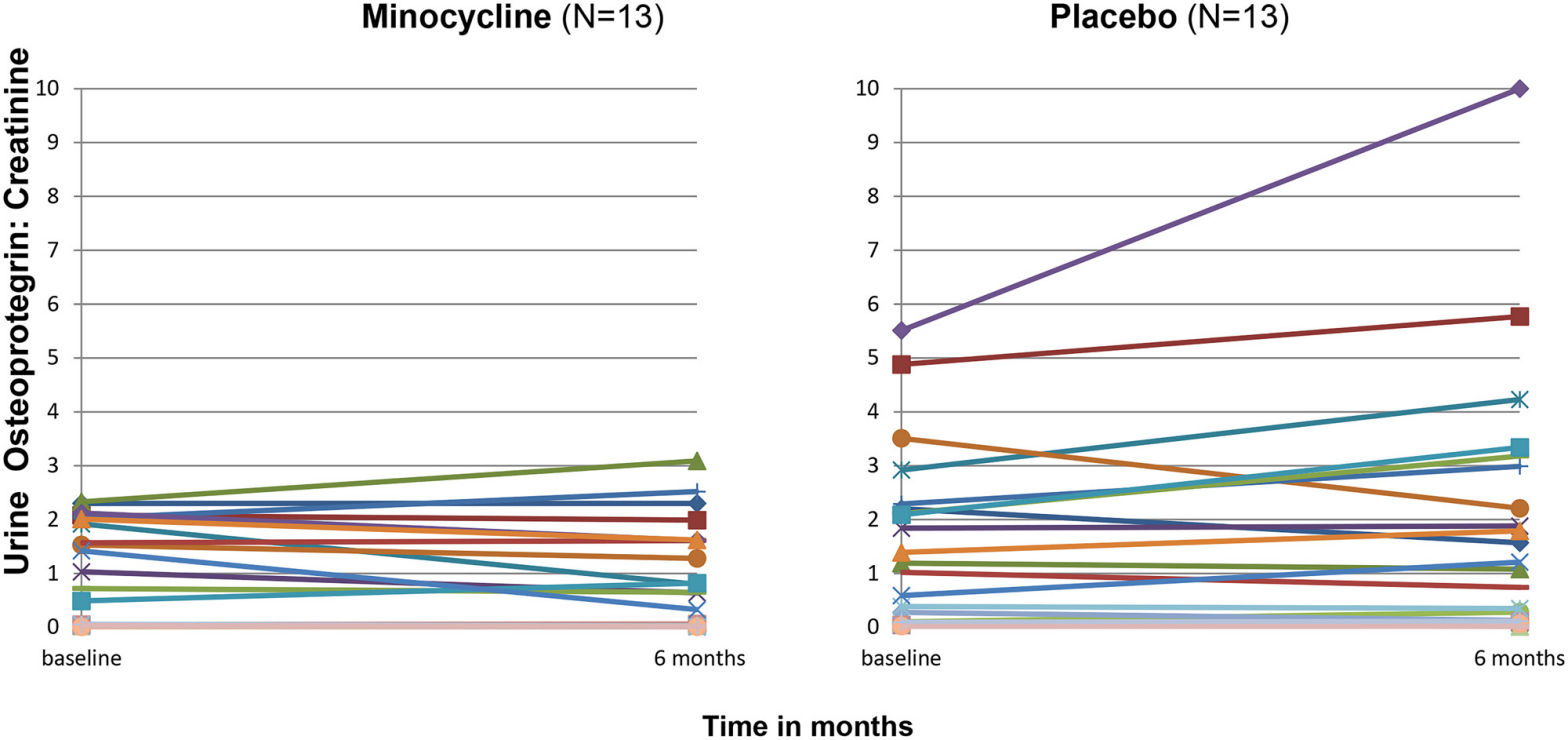
Spaghetti plots of the urine osteoprotegerin: Creatinine measurements of each patient in the study from baseline to 6 months (end of trial). Baseline urine values were not available for 3 subjects.

**Fig 7:**
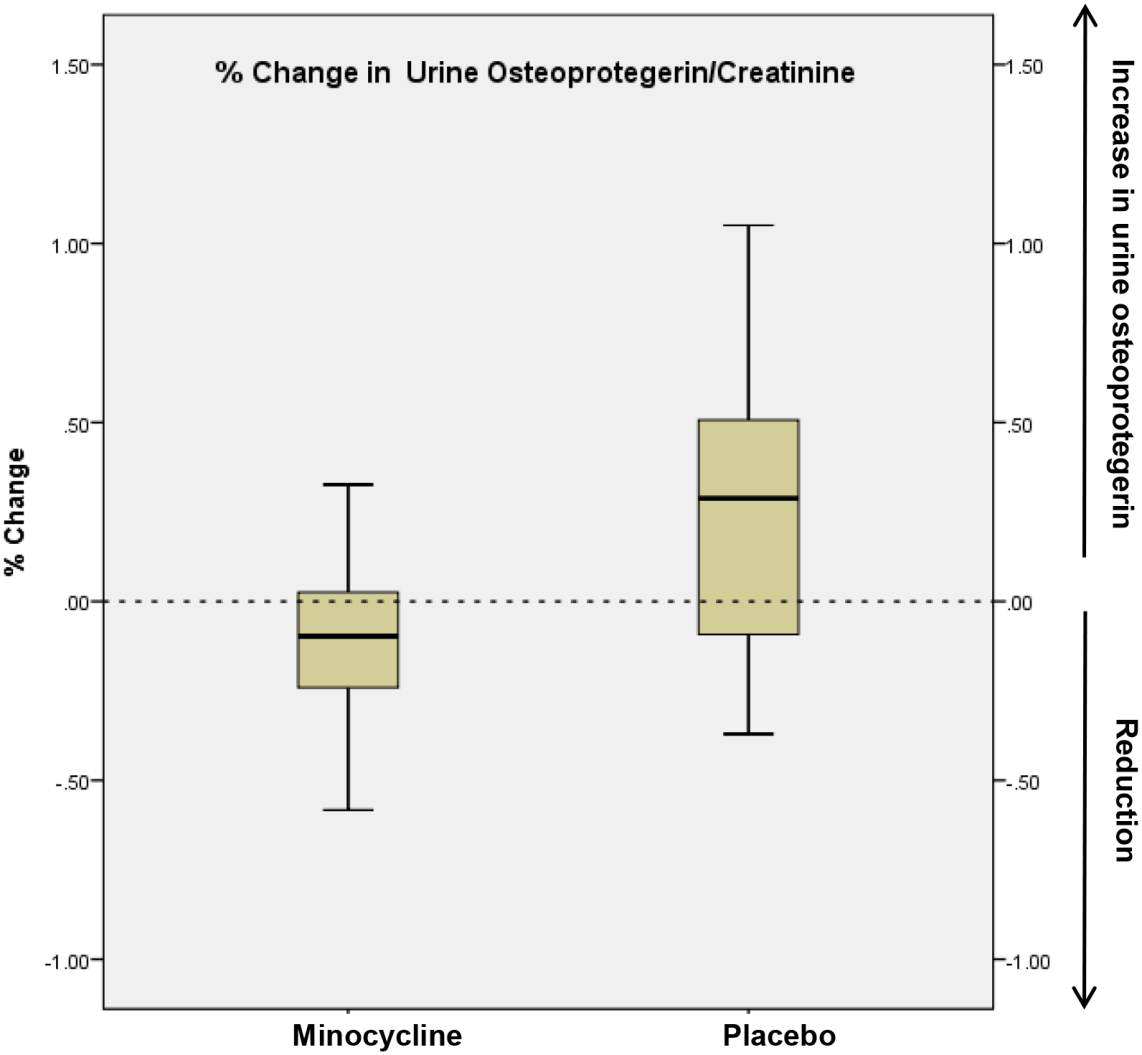
% change in 24-hour urine osteoprotegerin: creatinine measurements from baseline to 6 months in placebo and minocycline groups. The edges of the boxes represent the 25% and 75% percentiles, the middle lines represents the median, and the whiskers extend to the minimum and maximum values. The %change in urine osteoprotegerin: creatinine was significantly lower in the minocycline group vs. the placebo group (mean % change -0.09 vs. 0.25, P = 0.046).

We measured additional urine biomarkers to ascertain for biological proof-of-principle for a salutary effect of minocycline in patients with DN. Urine IL-6/creatinine (p = 0.03) and urine osteoprotegerin/creatinine (p = 0.046) measurements were higher at the end of the treatment period in the placebo group, but not in the minocycline group (Figs 4–7).

**Fig 8:**
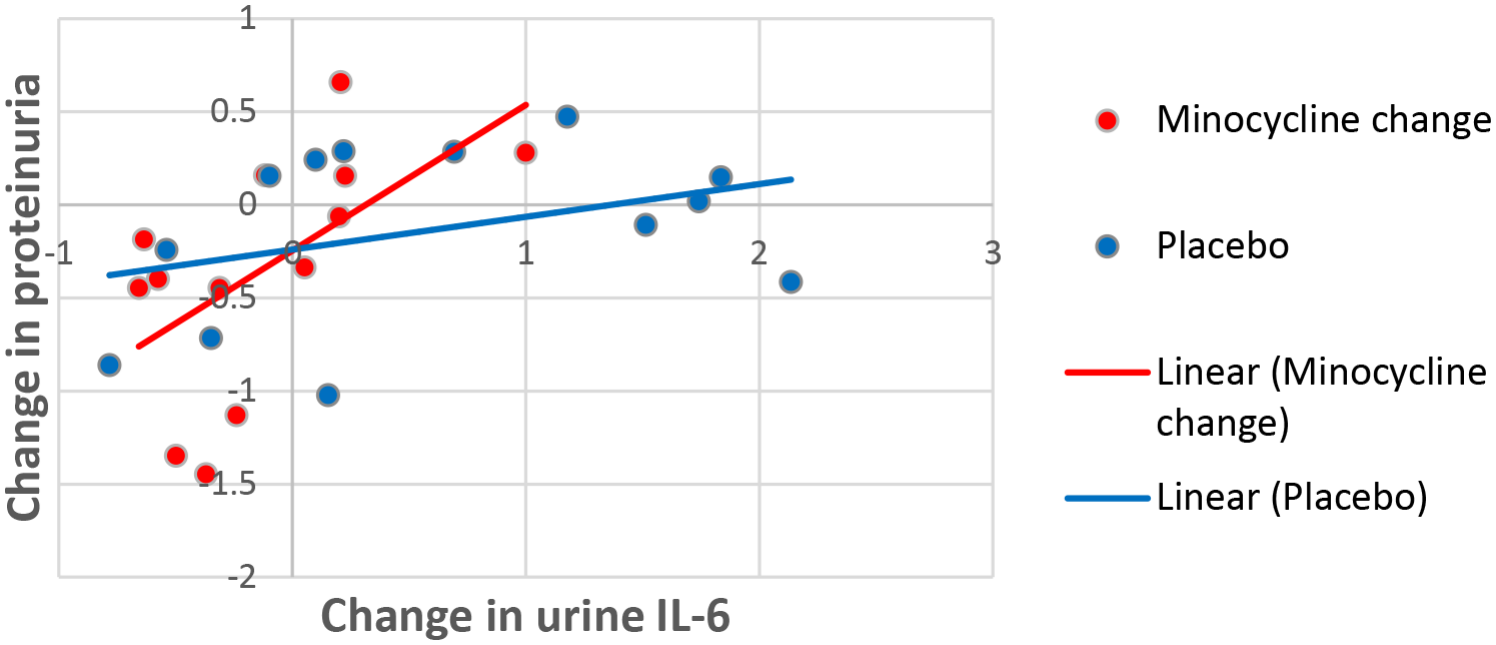
Relationship between changes in urine IL-6 and proteinuria in minocycline and placebo patients. The slope of the regression curve for placebo-treated patients is not different from zero (p> 0.05). The slope of the regression curve for minocycline-treated patients is different from zero (p< 0.05).

The relationship between the change in urine P:Cr and urine IL-6:Cr was evaluated (Fig 8). The slope of the regression curve for placebo-treated patients is not different from zero (p> 0.05). The slope of the regression curve for minocycline-treated patients is different from zero (p< 0.05). These data show that minocycline modifies the relationship between the change in urine IL-6: creatinine and urine P:Cr in a manner not observed in the relationship between these two in the placebo-treated group. Although the changes in osteoprotegerin in the minocycline and placebo groups were similar in direction to that observed for IL-6, the changes were less in magnitude. These changes did not meet statistical significance with regard to modifying the relationship between urine osteoprotegerin and protein as was observed for urine IL-6.

#### Change in creatinine clearance

**Fig 9:**
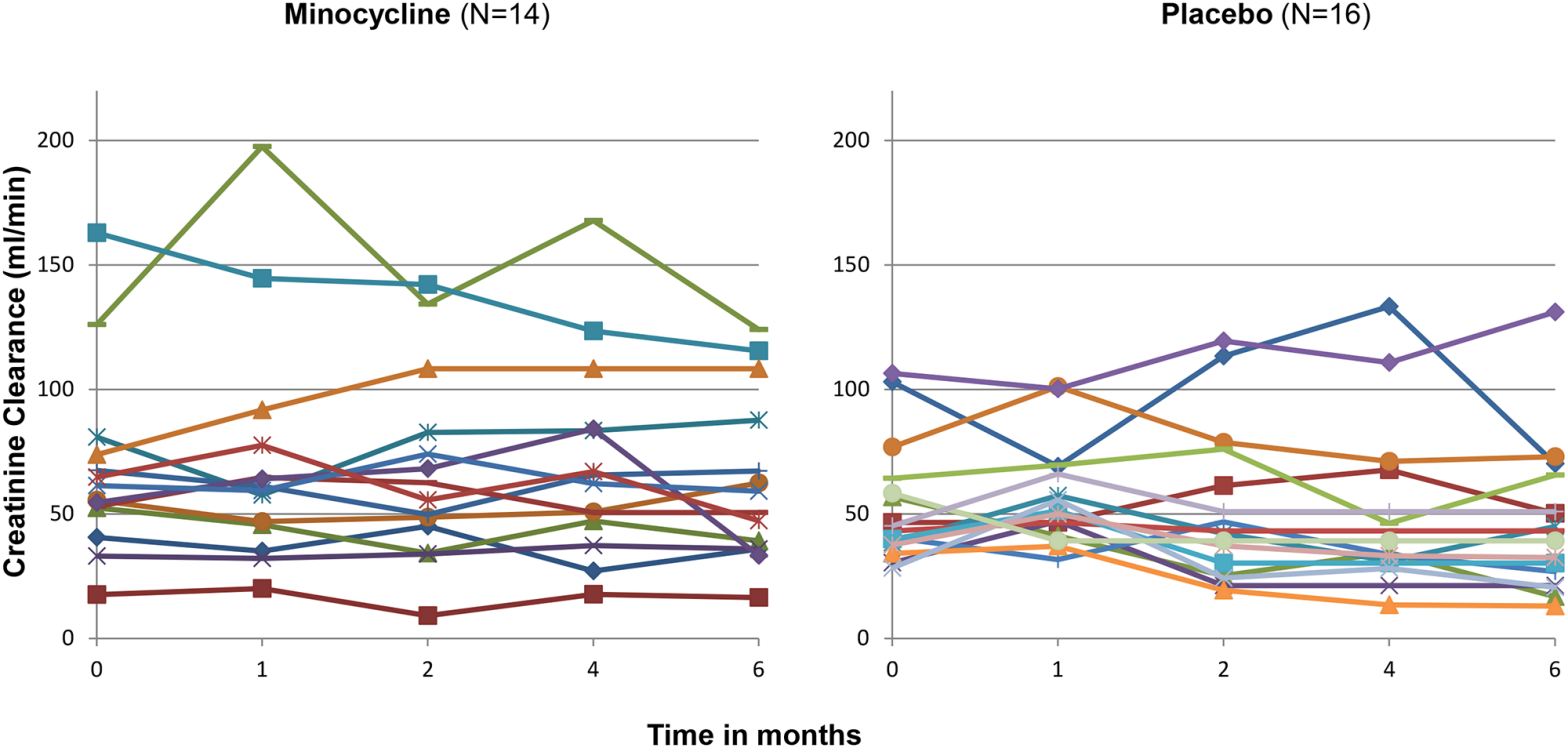
Spaghetti plots of the creatinine clearance measurements of each patient in the study over the course of the trial.

Although not statistically significant, the minocycline group tended to have a more stable creatinine clearance during the 6 month course of the study compared to the placebo group, even after adjustment for baseline creatinine clearance (Fig 9 and Table 4).

**Table 4:** Ratio of creatinine clearance (CrCl) in minocycline vs. placebo group, adjusted for baseline creatinine clearance.*

Time point	Estimated CrCl in the minocycline group / Estimated CrCl in the placebo group	P-value
1 month	0.92	0.44
2 months	1.04	0.69
4 months	1.22	0.07
6 months	1.20	0.10

*Estimated from a random intercept mixed-effects model for repeated measures.

### Safety assessments

Overall, minocycline was well-tolerated. Study medication was initially prescribed as one 100 mg tablet twice daily beginning on the day of randomization. Several of the patients who were initially randomized complained of nausea, vomiting, diarrhea, and/or dizziness in the first few days after the start of the medication. This necessitated a protocol change to begin tablets once daily for the first month, with an escalation as tolerated beginning month 2. One patient remained on a regimen of one tablet daily for the entire study. After unblinding, it was apparent that there was no difference in symptoms in patients taking minocycline compared to placebo (Table 5). Three patients became ANA-positive (titer 1:40) without lupus symptoms during the study. Study medication was discontinued in all upon discovery. Each had a termination visit that served as the final data in a last-observation-carry forward data analysis strategy. All were assigned placebo. ANA reverted to negative on follow-up in two of the three. One patient randomized to minocycline complained of yellow-tinged skin. RAASi protocol violations occurred in three patients in the minocycline group and one in the placebo group (dose changed or new drug started during the treatment period).

**Table 5:** Adverse and Serious Events (AE, SAE).

AE	Minocycline (n = 14)	Placebo (n = 16)
Gastrointestinal symptoms	3	2
Yellow Skin	1	0
Hospitalizations	5	3
End Stage Renal Disease	0	1
Dizziness	0	0
ANA	1	3

Serious adverse events involving eight hospitalizations occurred. Three patients receiving minocycline had five hospitalizations, but there were also three patients receiving placebo that had three hospitalizations. Among the minocycline-treated patients, one patient was hospitalized for shortness of breath and chest pain (separate admissions); one was hospitalized for chest pain; and one was hospitalized for two separate episodes of statin-related rhabdomyolysis. In the placebo-treated group, two patients were hospitalized for shortness of breath, and one patient had a single hospitalization for the development of end stage renal disease accompanied by accelerated hypertension, shortness of breath, anemia, and a vitreous hemorrhage.

## Discussion

Health care systems, overburdened treating the complications of a growing population of diabetic patients, including those with end stage renal disease, need inexpensive and safe adjunctive treatments with side-effect profiles different from the RAASi. Minocycline was tested as one potential therapy. The rationale for this was based on published pre-clinical and clinical work. Minocycline inhibited podocyte apoptosis in experimental DN, with virtually complete histological attenuation and mitigation of albuminuria[11]. It attenuated diabetic retinopathy in an animal model [10]. In a small Phase I/II six-month open-label pilot trial involving five patients with diabetic macular edema, minocycline improved best-corrected visual acuity, central retinal subfield thickness, central macular volume, and late leakage on fluorescein angiography [17]. In a clinical trial of 50 patients with Type 2 DM, minocycline was well-tolerated and improved peripheral neuropathy [18]. Two recently published studies reported on the potential of doxycycline, a related tetracycline antibiotic, as an anti-proteinuric agent for DN. In a randomized controlled trial involving 40 patients with DN, doxycycline lowered proteinuria, reaching statistical significance at 3 but not at 6 months [12]. In another pilot trial involving 35 diabetic subjects, doxycycline modestly reduced proteinuria from 888 ± 419 mg at baseline to 643 ± 386 mg after 2 months of treatment (p < 0.001)[13].

In the pilot study reported herein involving 30 patients with DN treated for 6 months, proteinuria declined in both the minocycline and placebo groups, numerically but not statistically significantly more in the minocycline group (6 month P:Cr: baseline P:Cr ratio 0.85 vs. 0.91 respectively, p = 0.27). However, two urinary biomarkers, IL-6 and osteoprotegerin (expressed corrected for urine creatinine concentration), were significantly reduced in the minocycline-treated patients. The biomarker changes suggest that minocycline may alter the pathobiology of and/or confer benefit to patients with DN.

Minocycline is a broad-spectrum semi-synthetic bacteriostatic tetracycline that has been used for decades. There has been a recent resurgence of clinical interest in minocycline, due to its potential for cytoprotection, particularly in neuronal cells [9]. The studies reported herein affirm modulation of cellular injury pathways.

Minocycline has been shown to have extra-renal cytoprotective properties in vivo and in vitro. Minocycline is an anti-oxidant [19], [20]. It has anti-inflammatory properties [21, 22] and is anti-apoptotic [23, 24]. Minocycline modulates inflammatory cell signaling pathways. Minocycline blocks NO-induced [21] and gentamicin-induced [25] mitogen-activated protein kinase (MAPK) activation in a stimulus-specific manner [26]. We showed that the podocyte p38MAPK pathway is activated in experimental DN, and is associated with downstream CREB phosphorylation and mesangial fibronectin accumulation [27, 28], suggesting a mechanism by which minocycline might modulate DN.

In the kidney, minocycline inhibited cytochrome C release and the up-regulation of p53 and Bax, and improved serum creatinine in a rat model of renal ischemia-reperfusion injury [29, 30]. Minocycline also decreased pathological vascular permeability after renal ischemia-reperfusion injury, and decreased tubulointerstitial inflammatory infiltrates, but not in the same spatial distribution [30]. In renal epithelial cells in vitro, minocycline protected against apoptosis induced by hypoxia, staurosporine, azide, and cis-platinum by suppressing Bax, outer membrane permeability, and cytochrome C release, and promoting the accumulation of anti-apoptotic Bcl-2 in mitochondria. Inhibition of Bcl-2 with antisense abolished this protective effect, implicating Bcl-2 directly in mediating the anti-apoptotic actions of minocycline [31].

Taken together, the data suggest that minocycline has biochemical properties that make it a promising anti-inflammatory and cytoprotective therapeutic agent to test for the treatment of diabetic complications. In this study, proteinuria improved in both the minocycline and the placebo-treated subjects. The small incremental decline in proteinuria that we observed in the minocycline group was not statistically significant compared to placebo. However, our small pilot study was only 14% powered to detect the difference seen. Based on the magnitude of change observed in this trial, a fully powered trial would require 138 patients per group to achieve 80% power to detect the difference observed.

In an effort to discern whether minocycline rendered a beneficial biological effect, we measured two biomarkers in the urine relevant to diabetic complications, IL-6 and osteoprotegerin. IL-6 is a 26 kD cytokine with a regulatory role in the generation of acute phase reactants and in chronic inflammation. While not a classical inflammatory disease, DN is now understood to involve chronic low-grade inflammation [32]. IL-6 is increased in the vitreous in experimental and clinical diabetic retinopathy [10, 33]. Urine and serum IL-6 increases as DN progresses [34]. Renal cortical IL-6 mRNA is higher in diabetic than control rat kidney and is associated with albuminuria [35]. Interstitial IL-6 mRNA in human diabetic kidney correlates with measures of tubulointerstitial injury [36]. While IL-6 is reportedly increased in both serum and urine in patients with DN and albuminuria, the serum and urinary levels do not correlate, suggesting independent phenomena [34],[37]. In patients with DN, high urine IL-6 levels predicted worse renal function over one year of follow-up [38]. Minocycline inhibited IL-6 in monocytes [39], in central nervous system parenchyma and/or infiltrating cells [40], and in ovarian cancer cells [41], but not in vitro in retinal microglia [10]. In ovarian cancer cells, minocycline also suppressed other members of the IL-6 receptor system, including IL-6R and gp130, signaling pathways STAT3 and ERK1/2, and the downstream target myeloid cell leukemia 1 [41]. Minocycline administered to patients over 72 hours suppressed the increment in serum IL-6 observed after acute ischemic stroke [42]. In this pilot trial, after 6 months of treatment, urine IL-6 was significantly lower in the minocycline compared to the placebo-treated group, consistent with previously identified effects on IL-6. In addition, minocycline modified the relationship between the change in urine IL-6 and urine protein in a manner not observed in the placebo group, suggesting its potential for conferring a salutary biological effect in DN and other diabetic complications.

Osteoprotegerin is a member of the tumor necrosis factor (TNF) receptor superfamily. Although initially identified as a regulator of bone resorption, osteoprotegerin is synthesized by mononuclear leukocytes and endothelial cells, and is expressed in the central nervous system, thyroid, heart, liver, lung, testes, ovaries, pancreas, adrenals, and kidneys. It serves as a soluble decoy receptor for the Receptor Activator of Nuclear Factor-B ligand (RANKL, aka Tumor Necrosis Factor Ligand Superfamily Member 11) and for TNF-related apoptosis-inducing ligand (TRAIL). It prevents osteoclast activation and bone resorption, and participates in immune regulation and cell survival. High plasma osteoprotegerin values have been linked to diabetic microvascular disease [43]. Plasma osteoprotegerin has been identified as an independent predictor of coronary artery disease in asymptomatic microalbuminuric Type 2 diabetic patients [44–46]. In a recently published study, high plasma osteoprotegerin levels identified a cohort of Type 1 diabetic patients with a higher risk of progression to ESRD. Furthermore, patients with higher levels had a more rapid decline in GFR [47]. In patients with systemic lupus erythematosus, high urine osteoprotegerin levels were associated with heavier proteinuria (> 500 mg/day) [48]. To our knowledge, our study is the first to measure the biomarker osteoprotegerin in the urine of DN patients, and the first to show that treatment with minocycline in DN can lower urinary osteoprotegerin excretion. Taken together with the data for urine IL-6, this data suggests that minocycline may confer renal cytoprotection in DN by altering the overall inflammatory milieu.

The interpretation of this study is subject to certain limitations. First, the generalizability of these results are limited to diabetic subjects with nephropathy with hyperkalemia limiting further RAASi therapy. Many of the patients were entered because there was no RAASi dose that could be used safely. Second, there was imbalance in the minocycline and placebo groups at baseline, with less severe proteinuria and a higher creatinine clearance in the minocycline group. While this difference was not statistically significant, it is possible that this imbalance may have contributed to the study’s outcome. Third, this pilot study was not adequately powered to detect the efficacy of minocycline to diminish proteinuria at the level observed, nor to test whether it preserves renal function. More subjects and a longer period of follow-up would be necessary in a fully powered study. Finally, although urine IL-6/creatinine and osteoprotegerin/creatinine declined more in the minocycline-treated group, and in the minocycline group there was a correlation between the change in urine IL-6 and the change in proteinuria not seen in the placebo group, it nevertheless is unclear if this reflected better permselectivity or a milieu of cytoprotection. Furthermore, these secondary analyses concerning biomarkers were exploratory only, and their actual significance requires additional prospective testing.

## Conclusion

In this small randomized, blinded, placebo-controlled pilot trial of minocycline as adjunctive treatment in DN, proteinuria improved in both the minocycline and the placebo-treated patients. Although there was a greater decline in the minocycline-treated group, this did not reach statistical significance. This study did demonstrate significant decrements in the urinary biomarkers IL-6 and osteoprotegerin. In addition, a relationship between the change in urine IL-6 and urine protein was observed in the minocycline group which was not observed in the placebo group, suggesting that this decrement was due to a salutary biological effect conferred by minocycline. No safety concerns were raised. Taken together, this pilot trial suggested biological efficacy signals and no safety concerns, supporting additional study of minocycline for the treatment of diabetic complications.

## Supporting Information

S1 CONSORT Checklist (PDF)

S1 Protocol (PDF)

S1 Dataset (XLSX)
