# Supplementary material for: Effects of Minocycline on Urine Albumin, Interleukin-6, and Osteoprotegerin in Patients with Diabetic Nephropathy: A Randomized Controlled Pilot Trial
Source: PLoS One. 2016 Mar 28;11(3):e0152357. doi: 10.1371/journal.pone.0152357 (PMC4809491; doi:10.1371/journal.pone.0152357)
Supplement: S1 Protocol — (PDF) [file pone.0152357.s002.pdf]

A. Specific Aims. Diabetic nephropathy (DN) remains a major source of mortality and morbidity in patients with Type 2 diabetes (T2DM). Although incident end stage renal disease (ESRD) from DN is declining, prevalence continues to rise (www.usrds.org). While ACEi and ARBs slow progression to ESRD, they do not regress DN, nor do they arrest progression. Maximal dosing is limited by side effects, including hyperkalemia, hypotension, and reduced glomerular filtration rate (GFR). ACEi and ARBs have been used simultaneously for therapeutic synergy in patients with non-diabetic renal disease [1]. However, the recent ONTARGET study, which included diabetic and non-diabetic patients with cardiovascular risk, failed to show improved cardiovascular outcomes from ACEi/ARB synergism. Instead, it showed increased renal functional decline [2], suggesting caution in the use of these agents together. Additional therapeutic agents including spironolactone [3, 4] and aliskerin [5] attenuate proteinuria in DN, but their major side effects are identical to the ACEi/ARBs. Affordable therapeutic agents with different side-effect profiles from those acting on the renin-angiotensin-aldosterone system (RAAS) are needed. Novel agents have recently either failed in a clinical trial (sulodexide, EJ Lewis, unpublished data presented at the meeting of the National Kidney Foundation, Spring 2008), or have not clearly been shown to be beneficial in secondary analyses (ruboxistaurin) [6]. The PI was lead author in a Phase 1 study to administer a connective tissue growth factor (CTGF) antibody to patients with microalbuminuria and either Type 1 or T2DM. The study showed safety, and data suggestive of, but not proving, anti-albuminuric efficacy [7]. A Phase 1 study with this agent in overt DN is in process. While the side-effect profile differs from medications inhibiting RAAS, the drug, if effective, will be expensive. A safe and inexpensive medication that doesn't lower GFR or blood pressure or raise serum K would be very useful as an adjunctive therapy for DN.

Minocycline is a semi-synthetic tetracycline whose broad cytoprotective properties have lately become apparent [8]. Recently, a seminal work by Isermann et al showed that minocycline inhibited podocyte apoptosis in experimental DN, with virtually complete histological attenuation and mitigation of albuminuria [9]. Minocycline also attenuated diabetic retinopathy in an animal model, via a mechanism involving inhibition of DM-induced cytokine-release and retinal apoptosis [10]. **Coupled to the protean reports of anti-oxidative, anti-fibrotic, and anti-apoptotic actions of minocycline in extra-renal tissues and cells, and some success in clinical trials in other inflammatory and degenerative diseases, these data support a rationale for testing minocycline in DN.**

Hypothesis: Minocycline will be a safe and effective anti-proteinuric adjunctive therapy to ACEi/ARBs in T2DM patients with proteinuria and eGFR $\geq$ 30 ml/min/m^2.

Specific Aims: Primary

- Assess minocycline safety in patients with DN and and eGFR $\geq$ 30 ml/min/m^2
- Determine whether minocycline at a dose of 100 mg po bid for 24 weeks (vs placebo) when added to a stable regimen of anti-proteinuric medications decreases proteinuria

Specific Aims: Secondary

- Compare the impact of minocycline therapy on proteinuria based on 24 hr, overnight, daytime, and random (spot) collections.
- Assess the change in cystatin C and creatinine clearance in minocycline vs placebo-treated patients at 24 weeks;
- These studies test a role for minocycline, a reasonably priced agent with a side-effect profile that differs from the RAAS inhibitors, in attenuating proteinuria in DN. If effective, these studies would support further direct testing of its efficacy in preserving renal function.

B. Background and significance

B1. Pathogenesis of DN and rationale for potential mitigation by minocycline. Until recently, DN was viewed almost completely as a mesangiocentric process, eg that progressive renal functional decline directly (and virtually solely) resulted from expanding mesangial matrix that occluded glomerular capillaries and ablated the capillary filtering surface area, resulting in organ failure [11-13]. In recent years, pathology in other renal compartments and their contribution to renal functional decline in DN have come to the fore. The PI reviewed the pathogenesis of DN [14-17]. Broadly, the clinical constellation of declining glomerular filtration rate (GFR) and proteinuria/albuminuria designated as DN represents a culmination of injury to all four renal compartments: vascular, mesangial, capillary wall, and tubulointerstitial. Acute and chronic hyperglycemia in the setting of systemic and intraglomerular hypertension, in genetically predisposed individuals, induces cascades of cellular pathology that broadly involve reactive oxygen species (ROS); alterations in cytokines, growth factors and their receptors, integrins and selectins; cytoskeletal disruption; extracellular matrix accretion; inflammation; disordered signal transduction; and disordered cell cycle regulation. These events induce endothelial injury leading arterial and arteriolar sclerosis and downstream ischemic change; mesangial expansion leading to glomerular capillary occlusion; podocyte effacement and apoptosis leading to proteinuria; and tubulointerstitial atrophy, apoptosis, and fibrosis, culminating in organ failure. **DN represents the culmination of glucose-induced pathologic cascades including oxidative stress, cytokines and growth factor elaboration, and their downstream signaling events in vessels, the glomerulus, and the tubulointerstitium, that lead to organ failure.**

B2. The podocyte in DN. Albuminuria is the earliest clinically detectable marker of DN. It is deemed so central that DN is classified by its severity. Albuminuria in DN reflects the sum of failure of the maintenance of permselectivity by the glomerular capillary wall, comprised of endothelial cells, the glomerular basement membrane (GBM), and the podocytes, coupled to a failure of downstream tubules to adequately reabsorb filtered albumin [18]. The podocyte has taken center stage in the pathogenesis of albuminuria not only in DN [9, 19-24], but in most proteinuric states [25, 26]. Proteinuria and albuminuria have come to be pathogenetically linked with progressive renal functional decline [9, 19-24, 27-29]. In DN, podocyte effacement, reflecting cytoskeletal injury, is a characteristic feature [30], and is ameliorated by ACE inhibitors [31]. Podocyte integrin expression is altered [32, 33]. Podocytes detach from

the GBM (unpublished observations), a process which would induce anoikis; their number and density are diminished [19, 21] and they are excreted in urine [34, 35]. Podocyte number inversely correlates with albuminuria [21, 36]. We showed numerous podocyte structural and signaling pathologies in experimental DN, including podocyte cytoskeletal disruption [19]; RhoA/cofilin and p38 MAPK dysregulation of podocyte actin cytoskeletal dynamics [37]; a decrement in podocyte density along the GBM [19]; mitigation of hypertrophy by ARB inhibition of p27kip1 in glucose-stimulated podocytes [27]; and down-regulation of expression of the podocyte slit membrane protein p-cadherin [24]. Recently, a seminal work by Isermann et al showed podocyte apoptosis in experimental DN, with virtually complete histological and "clinical" inhibition by mitigating apoptosis with minocycline [9]. **These data summarize the key role of podocytes in maintaining the glomerular filtration barrier and some of the major podocyte derangements in DN that contribute to albuminuria. Recent seminal data supporting the central role of apoptosis as a key feature of DN has been published, and experimental evidence for minocycline in mitigating apoptosis and albuminuria has been demonstrated [9]. If minocycline inhibits apoptosis in patients as it did in experimental DN, it may be a useful adjunct to ACEi/ARBs in the treatment of DN.**

B3. The biology of minocycline's cytoprotective properties. Minocycline is a broad-spectrum semi-synthetic bacteriostatic tetracycline that has been used for decades, most commonly for the treatment of acne and Lyme disease. Prior to the use of TNF inhibitors, it was commonly used for the treatment of rheumatoid arthritis [38, 39], although its mechanism of action was unclear. Recently, it has been suggested that minocycline confers cytoprotection via a variety of cellular actions. This has recently been reviewed [8]. Most experiments describing minocycline's cytoprotective properties have been performed in neuronal cells. Nevertheless, much of the demonstrated modulation of injury pathways are relevant to the pathobiology of DN. **Minocycline has anti-oxidant properties.** In cell-free models, minocycline is a radical scavenger with anti-oxidant efficacy on par with alpha-tocopherol [40]. It inhibits the generation of H_2O_2 resulting from activation of the NADPH oxidase and xanthine-xanthine oxidase systems [41]. In cell culture, minocycline inhibits COX-2 and iNOS mRNAs, but in vivo it failed to inhibit glutathione and NADPH or protect from brain injury in a mouse model of chemical brain injury [42-44]. **Minocycline has anti-inflammatory properties.** Minocycline reduced TNFα, IL-1β, and IL-6 in microglia incubated with human Ab42 amyloid peptide [45, 46]. Minocycline modulates inflammatory cell signaling pathways. The mitogen-activated protein kinases (MAPK) mediate signaling for many inflammatory mediators. Minocycline blocks NO-induced [47] and gentamicin [48] induced p38MAPK activation, but its ability to block p38MAPK is stimulus-specific [49]. We have shown that the p38MAPK pathway is activated in DN, and is associated with downstream CREB phosphorylation and mesangial fibronectin accumulation [28, 50], suggesting a potential means by which it might impact DN. These data support the possibility that minocycline-induced p38MAPK inhibition in the kidney might modulate mesangial expansion. **Minocycline has anti-apoptotic properties.** Minocycline attenuates mitochondrial permeability and swelling associated with calcium influx [51, 52] and inhibits the opening of the mitochondrial permeability transition pore [52], a key

step in apoptosis. Minocycline inhibits the release of otic hair cell mitochondrial cytochrome c after gentamicin [48] and inhibited lipopolysaccharide-induced IκBα degradation, thereby stabilizing NFκB in its bound state [49]. Minocycline has been shown to inhibit poly (ADP-ribose) polymerase-1 (PARP) in cultured cortical neurons injured with neurotoxins [53]. In an ischemia/reperfusion model in intact hearts and in cultured myocytes, minocycline reduced the expression of initiator caspases, increased the ratio of XIAP to SMAC/Diablo mRNA, prevented the release of cytochrome c and SMAC/Diablo, and limited infarct size, CPK release, myocardial cell apoptosis, and increased cell survival [54]. Thus, minocycline may attenuate podocyte apoptosis that occurs in DN.

The cytoprotective properties of minocycline are largely being tested clinically in neurologic diseases due to the penetration of minocycline across the blood-brain barrier [55, 56] coupled to its relatively favorable biological profile and its availability as an FDA-approved medication. Neurologic diseases in which minocycline is under consideration or under study for treatment in pilot trials in humans include multiple sclerosis (MS) [57, 58], Huntington’s disease [19], and Parkinson’s disease [59-61]. In MS [58], in a 6-month pilot trial involving 10 subjects, the mean number of gadolinium-enhancing brain lesions decreased from 1.38 to 0.22 ($p<0.03$). In Huntington’s disease, a pilot trial showed minocycline was well-tolerated [62], and a full scale clinical trial completed enrollment in May 2007, but results are not yet reported. Although in ALS, a small Phase I/II randomized, placebo-controlled feasibility trial showed tolerance of minocycline [63], a Phase II trial showed accelerated neurological progression in the treated subjects vs controls [64]. Despite these findings, a planned intervention with minocycline in MS is proceeding with caution in Canada.

Outside of the CNS, minocycline has been reported to be well-tolerated and beneficial in rheumatoid arthritis [38, 39] but not in scleroderma [65]. Minocycline decreased the oral corticosteroid requirement in patients with severe asthma [66]. An ongoing clinical trial for idiopathic pulmonary fibrosis (Clinical trials.gov registry number NCT00203697) has not yet reported results.

In the kidney, in a rat model of ischemia-reperfusion injury, minocycline inhibited cytochrome c release and the up-regulation of p53 and Bax, and improved the serum creatinine [67, 68]. Minocycline also decreased pathological vascular permeability after renal ischemia-reperfusion injury, and decreased tubulointerstitial inflammatory infiltrates, but not in the same spatial distribution [68]. In renal epithelial cells in vitro, minocycline protected against apoptosis induced by hypoxia, staurosporine, azide, and cisplatinum by suppressing Bax, outer membrane permeability, and cytochrome c release, and promoting the accumulation of anti-apoptotic Bcl-2 in mitochondria. Inhibition of Bcl-2 with antisense abolished this protective effect, implicating Bcl-2 directly in mediating the anti-apoptotic actions of minocycline [69].

Some initial data point to the potential for minocycline efficacy in DM. In an experimental model of DN in mice, minocycline inhibited podocyte apoptosis, albuminuria, glomerular and renal hypertrophy, and the expanded mesangial

extracellular matrix characteristic of DN [9]. Minocycline attenuated diabetic retinopathy in a mouse model [3]. Of note, in a recently published clinical pilot trial involving 35 diabetic subjects, doxycycline, which shares minocycline's cytoprotective properties, attenuated proteinuria from 888 ± 419 mg at baseline to 643 ± 386 mg after 2 months of doxycycline treatment ($p < 0.001$). Proteinuria increased during a doxycycline washout period to 1,021 ± 422 mg 2 months after doxycycline discontinuation. **Taken together, the data demonstrate that minocycline has biochemical properties that make it a promising therapeutic agent to test in DN.**

B4. Pharmacokinetics of minocycline. Minocycline is predominantly metabolized by the liver and only ~10% is excreted unchanged in urine. Its pharmacokinetics has been studied in patients with a broad range of renal impairment, from mild to ESRD, albeit in a relatively small number of subjects. No dosing change is recommended for renal impairment [70-72]. Absorption of minocycline may be impaired by antacids or iron-containing products. Its metabolism may be induced by anticonvulsants (other than gabapentin). Minocycline may decrease prothrombin activity, increasing bleeding tendency in patients taking anticoagulants. Minocycline may reduce the effectiveness of oral contraceptives, and raise the levels of lithium, digoxin, and theophylline [73]. **Minocycline can be administered to subjects with the range of renal function planned without the need to adjust dosing.**

B5. Side effects include dizziness, vertigo, headache, visual disturbances, confusion, intracranial hypertension (pseudotumor cerebri), SLE, rheumatoid arthritis, upset stomach, diarrhea, photosensitivity, fever, jaundice, sore throat, blue-gray skin discoloration with chronic use, nephrotoxicity from use of expired product, worsening of amyotrophic lateral sclerosis in a non-dose-dependent pattern [64]. Rarely, liver failure, azotemia, blood dyscrasias, intracranial hypertension, and black thyroid syndrome have been reported. May cause enamel hypoplasia in fetuses or young children, so is contraindicated in pregnant women and children under age 11 yrs. Tolerability is only moderate. In one study using 200 mg/day in patients with Parkinson's disease [59], side effects required drug discontinuation occurred in 23% of participants. Therefore, the sample size (see below) takes into account a potential drop-out rate of approximately 20%.

C. Methods. Study synopsis and assessment schedule

C1. Study Objectives. Is minocycline a safe and effective anti-proteinuric adjunctive therapy to ACEi/ARBs in T2DM patients with proteinuria and eGFR ≥ 30 ml/min/m^2 ?

Specific Aims: Primary

- Assess minocycline safety in patients with DN and eGFR ≥ 30 ml/min/m^2
- Determine whether minocycline at a dose of 100 mg po bid for 24 weeks (vs placebo) when added to a stable regimen of anti-proteinuric medications, decreases proteinuria

Specific Aims: Secondary

- Compare the impact of minocycline therapy on proteinuria based on 24 hour collections, overnight collections, and daytime collections;
- Assess the change in cystatin C and creatinine clearance in minocycline vs placebo-treated patients at 24 weeks.

C2. Design. This proof of concept study will employ a prospective, single center, randomized, placebo-controlled design. A total of 130 patients with DN will be enrolled. Patients will be randomized in two strata based on the mean 24 hour urine protein excretion at baseline, as follows: (a) < 3 g/24h and (b) ≥ 3 g/24h.

The study will consist of a 3-week screening period, a baseline visit (Day 0) during which the patients will be randomly assigned to the active drug (minocycline 100 mg po bid) or to matching placebo, a 24-week treatment period, a study completion visit at week 24, and a 2-month post-completion washout period with repeat urine and blood measures.

C3. Assessment schedule.

Procedure	Visit 0 Screen x Day -2x	Visit 0_2/ Enroll Day 0	Visit xmo Day 28 (+/- 5)	Visit 2mo Day 56 (+/- 5)	Visit 4mo Day xx6(+/- 5)	Visit 6mo Day x76 (+/- 5)	Washout Visit x Day 236
Overnight urine (creat, alb, prot)	x	x	x	x	x	x (x2)	x
Daytime urine (creat, alb, prot)			x	x	x	x	x
Random urine albumin/protein/creatinine			x	x	x	x	x
Adverse/Serious Adverse Events			x	x	x	x	x
Vital signs			x	x	x	x	x
CBC, Chemistry panel		x	x	x	x	x	x
Serum and urine cystatin C	x	x	x	x	x	x	x
Con Meds	x	x	x	x	x	x	x
Medical History (Full)	x						
Interim Medical History		x	x	x	x	x	x
Menstrual History	x	x	x	x	x	x	x
Patient Consent administer	x						
Physical Examination (Full)	x		x	x	x	x	x
Physical Examination (abbrev)		x	x	x	x	x	x
Randomization		x					
Serum beta-HCG	x					x	
Drug Assignment / Reconciliation	x	x	x	x	x	x	x
Subject Stipend	x	x	x	x	x	x	x
ANCA panel	x			x		x	

C4. Primary outcome: Safety and tolerability assessments. Safety assessments will consist of documenting concomitant medications and/or therapies; evaluating adverse events and serious adverse events; monitoring lab parameters including hematology, blood chemistries (including renal function, liver function, electrolytes, serum albumin), serum beta-HCG where appropriate, vital signs including blood pressure and weight, and assessments for edema. ANCA titers will be measured at baseline, and at 2 and 6 mos to ascertain subclinical vasculitis change. Vasculitis is a rare complication of minocycline therapy [74].

Criteria for the evaluation of safety:

- Comparison of pre-treatment to during treatment laboratory tests;
- Occurrence, severity, and duration of all adverse events and serious adverse events, with the investigator's assessment of causality
- Comparison of pre- and post-treatment physical examinations

C5. Primary Outcome: Proteinuria. The proteinuria primary outcome of this study will compare urine protein/creatinine of the baseline 24 hour collection (average of baseline and randomization) to the month 6 study end average collection (collected twice, at 3-7 days apart), in accordance with the most clinically relevant measurement. However, 24 hour urine collections will be performed as a split collection, separating the daytime from the overnight collection. 24 hour urine protein and creatinine will be calculated by adding the values of the component daytime and night-time collections. Study coordinators will provide written and verbal instructions on the proper collection technique, will provide labels for the patient to

designate the start and end time of each of the component collections, and will review this data when the collections containers are returned. The disadvantage of the increased complexity of this collection procedure is anticipated to be offset by the advantage of being able to directly compare the 24 hour result with daytime, night-time, and spot measurements of protein and creatinine with regard to longitudinal coefficient of variation and detection of differences according to the randomized comparison. Study eligibility will be based on >1 gm proteinuria on the full 24-hour urine collection obtained at the first screening visit (eg adding the urinary protein on the daytime and overnight collections). The primary outcome measure of proteinuria should attempt to ensure that changes in proteinuria observed are the consequences of the drug and are not a reflection of biological variability in proteinuria. Daytime collections have somewhat wide intra-individual reproducibility due to biologic variability associated with changes in posture, activity, protein intake, and hemodynamic changes. Estimated coefficient of variation (CV) of 24-hour urine protein measures in a clinical trial in patients receiving eculizimab for membranous nephropathy (Alexion Pharmaceuticals, unpublished data) and in a second clinical trial in patients receiving aminoguanidine for diabetic nephropathy (Alteon Pharmaceuticals, unpublished data) averaged 35%. During overnight collections, these changes are minimized, thereby potentially maximizing the likelihood that the differences observed are due to the randomized intervention. Low day to day variability in this overnight measure has been demonstrated in children [75]. In addition, in patients with a variety of non-diabetic renal diseases, the overnight urine protein/creatinine ratio correlated well with the 24-hour collection and was a more reliable predictor of decline in GFR and progression to ESRD than the 24-hour urinary protein excretion rate [76]. **Thus, as a secondary outcome of this study, the protocol will permit a comparison of daytime, night-time, and 24 hour urine protein/creatinine measurements to compare their relative CVs in the context of longitudinal assessments.**

Ruggenenti et al [76] reported that the most reliable measure of proteinuria and albuminuria are random (spot) protein/creatinine or albumin/creatinine ratios. The correlations reported in the Ruggenenti study [76] have not been universally observed in other publications. The urine protein/creatinine and or the urine albumin/creatinine ratio measured on overnight collection samples did not correlate well with the values obtained from 24-hour urine collections in one early study initially describing the usefulness of urine protein/creatinine ratios [77]. In another study of patients with renal disease, the second morning urine correlated better than the first morning urine with 24-hour urinary protein/creatinine measurements [78]. In healthy subjects, bed-rest collections for albumin showed less correlation with 24 hour urine measurements than daytime collections [79]. These divergent results may be understood in the context of the circadian rhythm of urinary protein, albumin and creatinine excretion in patients with and without renal disease. Circadian rhythm for proteinuria is noted for healthy people. Some suggest that it is also present in patients with renal disease [80], while others suggest that it is blunted [81]. Circadian rhythm of creatinine excretion is substantially blunted in patients with renal disease [80, 81]. These variations affect the urine protein/creatinine ratio measured at different times of the day.

The advantages of using the overnight collections are:

- Lower CV of the overnight collection
- Lower amount of protein/creatinine ratio, so a smaller absolute effect required to achieve outcome
- Greater likelihood that the intervention will have a direct effect (eg intervention unlikely to effect physiological processes that regulate postural proteinuria, hemodynamic changes associated with daytime activities, dietary protein intake)
- Best correlation with the functional outcomes of decline in GFR and progression to ESRD

The disadvantages of using overnight collections are:

- No prior study comparing the effect of an intervention on daytime vs overnight collections
- Questionable correlation of overnight ratio to the 24-hour protein excretion
- Overnight collection represents the lowest urine protein/creatinine ratio of the day

Given the advantages and disadvantages of each, we elected to use a familiar primary outcome (24 hour urine protein/creatinine) but contribute needed longitudinal data addressing other values (overnight, daytime, random) to determine which of the measures results in the lowest CV.

C6. Secondary Outcome: Proteinuria/Albuminuria/creatinine. Secondary proteinuria outcomes will compare the minocycline treated group with placebo and reflect the measurement of proteinuria in the different samples obtained. **Secondary proteinuria outcomes will compare changes in:**

- Urine protein/creatinine ratio in the complete collection (eg overnight + daytime).
- Urine protein/creatinine ratio in the daytime collection.
- Urine albumin/creatinine ratio in the overnight collection.
- Urine albumin/creatinine ratio in the complete collection (eg overnight + daytime).
- Urine albumin/creatinine ratio in the daytime collection.
- Urine protein/creatinine ratio on a random (spot) sample.
- Urine albumin/creatinine ratio on a random (spot) sample.

C7. Assess the change in cystatin C and creatinine clearance in minocycline vs placebo-treated patients at 24 weeks. The most clinically robust primary outcome for a study in DN is the classical doubling of serum creatinine, death and dialysis composite endpoint popularized by Bain et al for the Captopril trial [82]in Type 1 DN and used in subsequent DN trials [83, 84]. However, these studies require large numbers of subjects and long follow-up. Proteinuria has been advocated by nephrologists as a reasonable surrogate endpoint [85], though this approach cannot lead to registration from the point of view of the FDA. Thus, secondary endpoints for change in renal function would be useful for assessment of benefit. eGFR cannot be used to assess change in renal function in this analysis, since many of the subjects are likely to have mild impairment of renal function, and the eGFR equations are not validated for eGFR > 60 ml/min/m^2 [86]. Creatinine-based are imprecise due to the influence of diet and muscle mass [87], but are still used clinically in most centers due to their availability and their low cost. These measures will be used as secondary

analyses for renal function, and compared to changes in serum cystatin C levels [87]. Given the anticipated rate of change of renal function and the sample size, these assessments will be underpowered to detect differences, but may provide preliminary data for further evaluations if a trend is detected.

C8. Inclusion criteria:

- Clinical diagnosis of Type 2 DM and DN as described in the Family Investigation of Nephropathy and Diabetes Protocol [88]
- Baseline creatinine clearance $\geq$ 30 mL/min/1.73 m^2 (at first screening visit)
- Proteinuria ≥ 1.0 g/day (at first screening visit)
- Age ≥30 years
- BP at baseline <150/95 mm Hg (measured sitting after 10 min rest at first screening visit)
- Adequate hepatic function defined as total bilirubin < 1.5 x the upper limit of the normal range (ULN), AST (SGOT) and ALT (SGPT) < 2.5 x ULN.
- Patients taking ACEi, angiotensin receptor blockers (ARBs), aliskerin, spironolactone and/or diltiazem may be entered, but dosing may not change during the period of study or within 1 month prior to the first of the baseline proteinuria measurements.

C9. Exclusion criteria:

- NSAID (including COX-2 inhibitors) use > 3 tabs/week habitually
- Diagnosis of neurodegenerative diseases (Parkinson's disease, Huntington’s disease, multiple sclerosis, Alzheimer's disease, etc).
- Any unstable medical illness (unstable angina, advanced cancer, etc) over the last 30 days.
- History of liver disease (screening AST > 3 times the upper limit of normal)
- History of hematologic disease (screening white blood cell count less than 3,800/mm^3)
- History of systemic vasculitis or systemic lupus erythematosus
- Treatment with procainamide or hydralazine
- History of vestibular disease (excluding benign position vertigo)
- Pregnancy or lactation
- Allergy to tetracycline antibiotics
- Use of minocycline within thirty days of baseline visit
- Use of anti-epileptic medications other than gabapentin
- Use of lithium, digoxin, warfarin, other anticoagulants, and theophylline
- Limited mental capacity rendering the subject unable to provide written informed consent or comply with evaluation procedures
- History of recent alcohol or drug abuse or noncompliance with treatment or other experimental protocols
- Use of any investigational drug within 30 days prior to the baseline visit
- Women with the potential to become pregnant who are not willing to practice double-barrier birth control

D. Statistical methods

Sixty-five patients per treatment group will be recruited. This number accounts for a maximum of 20% drop-out rate (26 patients). A sample size of fifty-two patients per treatment group is sufficient to detect a 35% decrease in 24 hour urine protein in the minocycline group compared to the placebo group, with 90% power, assuming that the data is analyzed on a log-scale (natural base), a one-sided t-test at 0.025 confidence level and an intra subject CV of 50%. An estimate of the CV of 24-hour urine protein measures was made based on data available from a clinical trial in patients receiving eculizimab for membranous nephropathy (Alexion Pharmaceuticals, unpublished data) and confirmed with additional unpublished data based on urine collections from patients participating in a clinical trial of aminoguanidine in diabetic nephropathy (Alteon Pharmaceuticals). The study was powered to a 35% decrement in proteinuria in order to address both biological and statistical significance. Studies in other forms of glomerular disease have demonstrated that partial remissions prolong renal survival [89-92].

The change from baseline to week 24 in the 24 hour urine protein will be analyzed on the log-scale (natural base) using a linear model with treatment as factor and the baseline 24 hour urine protein (average of the screening and randomization) as covariate. The difference on the log-scale between minocycline and placebo and the corresponding two-sided 95% confidence interval will be calculated and the back-transformed % ratio and the corresponding two-sided 95% confidence interval will be presented.

The primary endpoint is the ratio: proteinuria/creatinine based on a 24 hour collection at month 6/proteinuria at baseline (average of two at each time point). The trial is considered a success if the one-sided t-test for the log-transformed primary endpoint of minocycline vs. placebo is significant at the 2.5% level. This test will only be performed at the end of the study.

E. Governance. The study will conducted under the oversight of the Los Angeles Biomedical Research Institute Compliance Office and the General Clinical Research Center (GCRC). The study will have its own monitor through the GCRC program, who will be otherwise independent of the study. Progress reports and adverse events will be reported quarterly in a cumulative fashion. Serious adverse events will be reported immediately.

References

1. Nakao, N., *Combination treatment of angiotensin-II receptor blocker and angiotensin-converting-enzyme inhibitor in non-diabetic renal disease (COOPERATE): a randomised controlled trial.* Lancet Neurol. **361**(117-24).
2. The ONTARGET Investigators, *Telmisartan, Ramipril, or Both in Patients at High Risk for Vascular Events10.1056/NEJMoa0801317.* N Engl J Med, 2008. **358**(15): p. 1547-1559.
3. Saklayen, M.G., et al., *Effects of additive therapy with spironolactone on proteinuria in diabetic patients already on ACE inhibitor or ARB therapy: results of a randomized, placebo-controlled, double-blind, crossover trial.* J Investig Med, 2008. **56**(4): p. 714-9.
4. Chrysostomou, A., et al., *Double-blind, placebo-controlled study on the effect of the aldosterone receptor antagonist spironolactone in patients who have persistent proteinuria and are on long-term angiotensin-converting enzyme inhibitor therapy, with or without an angiotensin II receptor blocker.* Clin J Am Soc Nephrol, 2006. **1**(2): p. 256-62.
5. Parving, H.H., et al., *Aliskiren combined with losartan in type 2 diabetes and nephropathy.* N Engl J Med, 2008. **358**(23): p. 2433-46.
6. Tuttle, K.R., et al., *The Effect of Ruboxistaurin on Nephropathy in Type 2 Diabetes10.2337/diacare.28.11.2686.* Diabetes Care, 2005. **28**(11): p. 2686-2690.
7. Adler, S.S., S; Williams,ME; Arauz,C; Bolton,WK; Lee,T; Coker,G; Sewell KL:, *Dose-escalation phase I study of FG-3019 anti-connective tissue growth factor (CTGF) monoclonal antibody, in subjects with type I/II diabetes mellitus and microalbuminuria.* J Am Soc Nephrol, 2006. **17**: p. 157A.
8. Jordan, J., et al., *Minocycline and cytoprotection: shedding new light on a shadowy controversy.* Curr Drug Deliv, 2007. **4**(3): p. 225-31.
9. Isermann, B., et al., *Activated protein C protects against diabetic nephropathy by inhibiting endothelial and podocyte apoptosis.* Nat Med, 2007. **13**(11): p. 1349-58.
10. Krady, J.K., et al., *Minocycline Reduces Proinflammatory Cytokine Expression, Microglial Activation, and Caspase-3 Activation in a Rodent Model of Diabetic Retinopathy10.2337/diabetes.54.5.1559.* Diabetes, 2005. **54**(5): p. 1559-1565.
11. Osterby, R., *Early phases in the development of diabetic glomerulopathy.* Acta Med Scand Suppl, 1974. **574**: p. 3-82.
12. Dalla Vestra, M. and P. Fioretto, *Diabetic nephropathy: renal structural studies in type 1 and type 2 diabetic patients.* International Congress Series. The metabolic syndrome: diabetes, obesity, hyperlipidemia and hypertension. Proceedings of the 8th European Symposium on Metabolism, 2003. **1253**: p. 163-169.
13. Mauer, S.M., et al., *Structural-functional relationships in diabetic nephropathy.* J Clin Invest, 1984. **74**(4): p. 1143-55.

14. Adler, S., *Structure-function relationships in diabetic nephropathy: lessons and limitations.* Kidney Int Suppl, 1997. **60**: p. S42-5.
15. Adler, S., *Structure-function relationships associated with extracellular matrix alterations in diabetic glomerulopathy.* J Am Soc Nephrol, 1994. **5**(5): p. 1165-72.
16. Adler, S., *Diabetic nephropathy: Linking histology, cell biology, and genetics.* 2004. **66**(5): p. 2095-2106.
17. Adler, S., C. Nast, and A. Artishevsky, *Diabetic nephropathy: pathogenesis and treatment.* Annu Rev Med, 1993. **44**: p. 303-15.
18. Russo, L.M., et al., *Renal processing of albumin in diabetes and hypertension in rats: possible role of TGF-beta1.* Am J Nephrol, 2003. **23**(2): p. 61-70.
19. Dai, T., et al., *Glucose and diabetes: effects on podocyte and glomerular p38MAPK, heat shock protein 25, and actin cytoskeleton.* Kidney Int, 2006. **69**(5): p. 806-14.
20. Macconi, D., et al., *Pathophysiologic Implications of Reduced Podocyte Number in a Rat Model of Progressive Glomerular Injury10.2353/ajpath.2006.050398.* Am J Pathol, 2006. **168**(1): p. 42-54.
21. Pagtalunan, M.E., et al., *Podocyte loss and progressive glomerular injury in type II diabetes.* J Clin Invest, 1997. **99**(2): p. 342-8.
22. White, K.E., et al., *Podocyte Number in Normotensive Type 1 Diabetic Patients With Albuminuria 10.2337/diabetes.51.10.3083.* Diabetes, 2002. **51**(10): p. 3083-3089.
23. Wolf, G., S. Chen, and F.N. Ziyadeh, *From the Periphery of the Glomerular Capillary Wall Toward the Center of Disease: Podocyte Injury Comes of Age in Diabetic Nephropathy 10.2337/diabetes.54.6.1626.* Diabetes, 2005. **54**(6): p. 1626-1634.
24. Xu, Z.-G., et al., *P-Cadherin is decreased in diabetic glomeruli and in glucose-stimulated podocytes in vivo and in vitro studies 10.1093/ndt/gfh642.* Nephrol. Dial. Transplant., 2005. **20**(3): p. 524-531.
25. White, K.E. and R.W. Bilous, *Structural alterations to the podocyte are related to proteinuria in type 2 diabetic patients 10.1093/ndt/gfh129.* Nephrol. Dial. Transplant., 2004. **19**(6): p. 1437-1440.
26. Pavenstadt, H., *Roles of the podocyte in glomerular function.* Am J Physiol Renal Physiol, 2000. **278**(2): p. F173-179.
27. Xu, Z.-G., et al., *Angiotensin II receptor blocker inhibits p27Kip1 expression in glucose-stimulated podocytes and in diabetic glomeruli.* 2005. **67**(3): p. 944-952.
28. Kang, S.W., et al., *Role of 12-lipoxygenase in the stimulation of p38 mitogen-activated protein kinase and collagen alpha5(IV) in experimental diabetic nephropathy and in glucose-stimulated podocytes.* J Am Soc Nephrol, 2003. **14**(12): p. 3178-87.
29. Susztak, K., et al., *Glucose-Induced Reactive Oxygen Species Cause Apoptosis of Podocytes and Podocyte Depletion at the Onset of Diabetic Nephropathy 10.2337/diabetes.55.01.06.db05-0894.* Diabetes, 2006. **55**(1): p. 225-233.
30. Bjorn, S.F., et al., *Glomerular epithelial foot processes and filtration slits in IDDM patients.* Diabetologia, 1995. **38**(10): p. 1197-204.
31. Mifsud, S.A., et al., *Podocyte foot process broadening in experimental diabetic nephropathy: amelioration with renin-angiotensin blockade.* Diabetologia, 2001. **44**(7): p. 878-82.
32. Chen, H.C., et al., *Altering expression of alpha3beta1 integrin on podocytes of human and rats with diabetes.* Life Sci, 2000. **67**(19): p. 2345-53.

33. Kitsiou, P.V., et al., *Glucose-induced changes in integrins and matrix-related functions in cultured human glomerular epithelial cells.* Am J Physiol Renal Physiol, 2003. **284**(4): p. F671-9.
34. Nakamura, T., et al., *Urinary excretion of podocytes in patients with diabetic nephropathy 10.1093/ndt/15.9.1379.* Nephrol. Dial. Transplant., 2000. **15**(9): p. 1379-1383.
35. Nakamura, T., et al., *Effect of the antiplatelet drug dilazep dihydrochloride on urinary podocytes in patients in the early stage of diabetic nephropathy 10.2337/diacare.23.8.1168.* Diabetes Care, 2000. **23**(8): p. 1168-1171.
36. Meyer, T.W., *Podocyte number predicts long-term urinary albumin excretion in Pima Indians with Type II diabetes and microalbuminuria.* 1999. **42**: p. 1341-4.
37. Chamberlin, M., *RhoGTPases and actin depolymerizing protein cofilin in diabetic nephropathy (DN).* J Am Soc Nephrol, 2007. **18.**: p. 653A.
38. Tilley, B.C., et al., *Minocycline in Rheumatoid Arthritis: A 48-Week, Double-Blind, Placebo-Controlled Trial.* Ann Intern Med, 1995. **122**(2): p. 81-89.
39. Kloppenburg, M., *Minocycline in active rheumatoid arthritis. A double-blind, placebo-controlled trial.* Arthritis and Rheum, 1994. **37**: p. 629-36.
40. Kraus, R.L., et al., *Antioxidant properties of minocycline: neuroprotection in an oxidative stress assay and direct radical-scavenging activity.* J Neurochem, 2005. **94**(3): p. 819-27.
41. Choi, S.H., *Inhibition of thrombin-induced microglial activation and NADPH oxidase by minocycline protects dopaminergic neurons in the substantia nigra in vivo.* Jl of Neurochem, 2005. **95**: p. 1755-65.
42. Fernandez-Gomez, F.J., et al., *Minocycline fails to protect cerebellar granular cell cultures against malonate-induced cell death.* Neurobiol Dis, 2005. **20**(2): p. 384-91.
43. Fernandez-Gomez, F.J., et al., *Malonate induces cell death via mitochondrial potential collapse and delayed swelling through an ROS-dependent pathway.* Br J Pharmacol, 2005. **144**(4): p. 528-37.
44. Jordan, J., et al., *Superoxide anions mediate veratridine-induced cytochrome c release and caspase activity in bovine chromaffin cells.* Br J Pharmacol, 2002. **137**(7): p. 993-1000.
45. Seabrook, T.J., et al., *Minocycline affects microglia activation, Abeta deposition, and behavior in APP-tg mice.* Glia, 2006. **53**(7): p. 776-82.
46. Familian, A., et al., *Inhibitory effect of minocycline on amyloid beta fibril formation and human microglial activation.* Glia, 2006. **53**(3): p. 233-40.
47. Lin, S., et al., *Minocycline blocks nitric oxide-induced neurotoxicity by inhibition p38 MAP kinase in rat cerebellar granule neurons.* Neurosci Lett, 2001. **315**(1-2): p. 61-4.
48. Wei, X., et al., *Minocycline prevents gentamicin-induced ototoxicity by inhibiting p38 MAP kinase phosphorylation and caspase 3 activation.* Neuroscience, 2005. **131**(2): p. 513-21.
49. Nikodemova, M., I.D. Duncan, and J.J. Watters, *Minocycline exerts inhibitory effects on multiple mitogen-activated protein kinases and IkappaBalpha degradation in a stimulus-specific manner in microglia.* J Neurochem, 2006. **96**(2): p. 314-23.
50. Kang, S.W., et al., *p38 MAPK and MAPK kinase 3/6 mRNA and activities are increased in early diabetic glomeruli.* Kidney Int, 2001. **60**(2): p. 543-52.
51. Zhu, S., et al., *Minocycline inhibits cytochrome c release and delays progression of amyotrophic lateral sclerosis in mice.* Nature, 2002. **417**(6884): p. 74-8.
52. Fernandez-Gomez, F.J., et al., *Involvement of mitochondrial potential and calcium buffering capacity in minocycline cytoprotective actions.* Neuroscience, 2005. **133**(4): p. 959-67.

53. Alano, C.C., et al., *Minocycline inhibits poly(ADP-ribose) polymerase-1 at nanomolar concentrations.* Proc Natl Acad Sci U S A, 2006. **103**(25): p. 9685-90.
54. Scarabelli, T.M., et al., *Minocycline inhibits caspase activation and reactivation, increases the ratio of XIAP to smac/DIABLO, and reduces the mitochondrial leakage of cytochrome C and smac/DIABLO 10.1016/j.jacc.2003.09.050.* J Am Coll Cardiol, 2004. **43**(5): p. 865-874.
55. Blum, D., et al., *Clinical potential of minocycline for neurodegenerative disorders.* Neurobiol Dis, 2004. **17**(3): p. 359-66.
56. Zemke, D. and A. Majid, *The potential of minocycline for neuroprotection in human neurologic disease.* Clin Neuropharmacol, 2004. **27**(6): p. 293-8.
57. Zhang, Y., et al., *Pilot study of minocycline in relapsing-remitting multiple sclerosis.* Can J Neurol Sci, 2008. **35**(2): p. 185-91.
58. Metz, L.M., *Minocycline reduces gadolinium-enhancing magnetic resonance imaging lesions in multiple sclerosis.* Ann of Neurology, 2004. **55**: p. 5.
59. *A pilot clinical trial of creatine and minocycline in early Parkinson disease: 18-month results.* Clin Neuropharmacol, 2008. **31**(3): p. 141-50.
60. LeWitt, P.A. and D.C. Taylor, *Protection against Parkinson's disease progression: clinical experience.* Neurotherapeutics, 2008. **5**(2): p. 210-25.
61. Thomas, M. and W.D. Le, *Minocycline: neuroprotective mechanisms in Parkinson's disease.* Curr Pharm Des, 2004. **10**(6): p. 679-86.
62. Group, H.S., *Minocycline safety and tolerability in Huntington disease.* Neurology, 2004. **63**: p. 547-9.
63. Gordon, P.H., et al., *Placebo-controlled phase I/II studies of minocycline in amyotrophic lateral sclerosis.* Neurology, 2004. **62**(10): p. 1845-1847.
64. Gordon, P.H., *Efficacy of minocycline in patients with amyotrophic lateral sclerosis: a phase III randomised trial.* Lancet Neurol, 2007. **6**: p. 1045-53.
65. MD, M., *Minocycline is not effective in systemic sclerosis: results of an open-label multicenter trial.* Arthritis and Rheum, 2004. **50**: p. 553-7.
66. Ammar, D., *Minocycline treatment results in reduced oral steroid requirements in adult asthma.* Allergy Asthma Proc, 2008. **3**: p. 286-94.
67. Kelly, K.J., et al., *Minocycline inhibits apoptosis and inflammation in a rat model of ischemic renal injury.* Am J Physiol Renal Physiol, 2004. **287**(4): p. F760-766.
68. Sutton, T.A., et al., *Minocycline reduces renal microvascular leakage in a rat model of ischemic renal injury.* Am J Physiol Renal Physiol, 2005. **288**(1): p. F91-7.
69. Wang, J., et al., *Minocycline up-regulates Bcl-2 and protects against cell death in mitochondria.* J Biol Chem, 2004. **279**(19): p. 19948-54.
70. Sklenar, I., P. Spring, and L. Dettli, *One-dose and multiple-dose kinetics of minocycline in patients with renal disease.* Agents Actions, 1977. **7**(3): p. 369-77.
71. Welling, P.G., et al., *Pharmacokinetics of minocycline in renal failure.* Antimicrob Agents Chemother, 1975. **8**(5): p. 532-7.
72. Heaney, D. and G. Eknoyan, *Minocycline and doxycycline kinetics in chronic renal failure.* Clin Pharmacol Ther, 1978. **24**(2): p. 233-9.
73. Saivin, S. and G. Houin, *Clinical pharmacokinetics of doxycycline and minocycline.* Clin Pharmacokinet, 1988. **15**(6): p. 355-66.
74. Pelletier, F., et al., *Minocycline-induced cutaneous polyarteritis nodosa with antineutrophil cytoplasmic antibodies.* Eur J Dermatol, 2003. **13**(4): p. 396-8.

75. Yoshimoto, M., et al., *Evaluation of variability of proteinuria indices.* Pediatr Nephrol, 1990. **4**(2): p. 136-9.
76. Ruggenenti, P., et al., *Cross sectional longitudinal study of spot morning urine protein:creatinine ratio, 24 hour urine protein excretion rate, glomerular filtration rate, and end stage renal failure in chronic renal disease in patients without diabetes.* Bmj, 1998. **316**(7130): p. 504-9.
77. Ginsberg, J.M., *Use of single voided urine samples to estimate quantitative proteinuria.* NEJM, 1983. **309**: p. 1543-6.
78. Kristal, B., et al., *Estimation of quantitative proteinuria by using the protein-creatinine ratio in random urine samples.* Am J Nephrol, 1988. **8**(3): p. 198-203.
79. Kouri, T., et al., *Reference intervals for the markers of proteinuria with a standardised bed-rest collection of urine.* Clin Chem Lab Med, 2001. **39**(5): p. 418-25.
80. Koopman, M.G., et al., *Circadian Rhythm of Proteinuria: Consequences of the Use of Urinary Protein:Creatinine Ratios.* Nephrol. Dial. Transplant., 1989. **4**(1): p. 9-14.
81. Buzio, C., et al., *Circadian rhythm of proteinuria in normal subjects but not in patients with glomerulonephritis.* Ann Clin Res, 1987. **19**(1): p. 30-3.
82. Bain, R., et al., *A controlled clinical trial of angiotensin-converting enzyme inhibition in type I diabetic nephropathy: study design and patient characteristics. The Collaborative Study Group.* J Am Soc Nephrol, 1992. **3**(4 Suppl): p. S97-103.
83. Rodby, R.A., et al., *The Irbesartan type II diabetic nephropathy trial: study design and baseline patient characteristics. For the Collaborative Study Group.* Nephrol Dial Transplant, 2000. **15**(4): p. 487-97.
84. Brenner, B.M., et al., *Effects of Losartan on Renal and Cardiovascular Outcomes in Patients with Type 2 Diabetes and Nephropathy 10.1056/NEJMoa011161.* N Engl J Med, 2001. **345**(12): p. 861-869.
85. Stevens, L.A., T. Greene, and A.S. Levey, *Surrogate End Points for Clinical Trials of Kidney Disease Progression 10.2215/CJN.00600206.* Clin J Am Soc Nephrol, 2006. **1**(4): p. 874-884.
86. Stevens, L.A., et al., *Assessing Kidney Function -- Measured and Estimated Glomerular Filtration Rate 10.1056/NEJMra054415.* N Engl J Med, 2006. **354**(23): p. 2473-2483.
87. Stevens, L.A., et al., *Estimating GFR using serum cystatin C alone and in combination with serum creatinine: a pooled analysis of 3,418 individuals with CKD.* Am J Kidney Dis, 2008. **51**(3): p. 395-406.
88. Knowler, W.C., et al., *The Family Investigation of Nephropathy and Diabetes (FIND): design and methods.* J Diabetes Complications, 2005. **19**(1): p. 1-9.
89. Chen, Y.E., et al., *Value of a complete or partial remission in severe lupus nephritis.* Clin J Am Soc Nephrol, 2008. **3**(1): p. 46-53.
90. Reich, H.N., et al., *Remission of proteinuria improves prognosis in IgA nephropathy.* J Am Soc Nephrol, 2007. **18**(12): p. 3177-83.
91. Troyanov, S., et al., *Focal and segmental glomerulosclerosis: definition and relevance of a partial remission.* J Am Soc Nephrol, 2005. **16**(4): p. 1061-8.
92. Troyanov, S., et al., Idiopathic membranous nephropathy: definition and relevance of a partial remission. Kidney Int, 2004. **66**(3): p. 1199-205.
